# Phytohormonal dynamics in the abscission zone of Korla fragrant pear during calyx abscission: a visual study

**DOI:** 10.3389/fpls.2024.1452072

**Published:** 2024-10-08

**Authors:** Lingling Zheng, Yue Wen, Yan Lin, Jia Tian, Junjie Shaobai, Zhichao Hao, Chunfeng Wang, Tianyu Sun, Lei Wang, Chen Chen

**Affiliations:** ^1^ College of Horticulture, Xinjiang Agricultural University, Urumqi, Xinjiang, China; ^2^ Korla Fragrant Pear Research Centre, Korla, Xinjiang, China

**Keywords:** Korla fragrant pear, calyx abscission, abscission zone, microstructure, phytohormone, spatial distribution, MALDI-MSI technique

## Abstract

**Introduction:**

Phytohormones play a crucial role in regulating the abscission of plant organs and tissues.

**Methods:**

In this study, the ultrastructure of the sepals of Korla fragrant pears was observed using a transmission electron microscope, and high-performance liquid and gas chromatography were used to analyze the dynamic changes of phytohormones in the abscission zone during the calyx abscission process of Korla fragrant pears, and mass spectrometry imaging was applied to ascertain the spatial distribution of phytohormones.

**Results:**

The results revealed that the mitochondria in the abscission zone of the decalyx fruits were regularly distributed around the cell wall, and the chloroplasts were moderately present. In contrast, in the persistent calyx fruit, the corresponding parts of the abscission zone showed a scattered distribution of mitochondria within the cells, and there was a higher number of chloroplasts, which also contained starch granules inside. Mass spectrometry imaging revealed that ABA was enriched in the abscission zone of the decalyx fruit, and their ionic signal intensities were significantly stronger than those of the persistent calyx fruit. However, the ionic signal intensities of Indole-3-acetic acid (IAA) and Gibberellin A3 (GA_3_) of the persistent calyx fruit were significantly stronger than those in the abscission zone of the decalyx fruit and were concentrated in the persistent calyx fruit. 1-Aminocyclopropanecarboxylic Acid (ACC) did not show distinct regional distribution in both the decalyx and persistent calyx fruits. Furthermore, before the formation of the abscission zone, the levels of IAA, GA_3_, and zeatin (ZT) in the abscission zone of the decalyx fruits were significantly lower than those in the persistent calyx fruits by 37.9%, 57.7%, and 33.0%, respectively, while the levels of abscisic acid (ABA) and ethylene (ETH) were significantly higher by 21.9% and 25.0%, respectively. During the formation of the abscission zone, the levels of IAA, GA_3_, and ZT in the abscission zone of the decalyx fruits were significantly lower than those in the persistent calyx fruits by 41.7%, 71.7%, and 24.6%, respectively, while the levels of ABA and ETH were significantly higher by 15.2% and 80.0%, respectively. After the formation of the abscission zone, the levels of IAA and GA_3_ in the abscission zone of the decalyx fruits were lower than those in the persistent calyx fruits by 20.8% and 47.8%, respectively, while the levels of ABA and ETH were higher by 271.8% and 26.9%, respectively. In summary, during the calyx abscission process of Korla fragrant pears, IAA and GA_3_ in the abscission zone inhibited abscission, while ABA and ETH promoted calyx abscission. These research findings enrich the understanding of the regulatory mechanism of plant hormones on calyx abscission and provide a theoretical basis for the study of exogenous plant growth regulators for regulating calyx abscission in Korla fragrant pear.

## 1 Introduction

Plant organ or tissue abscission is a complex physiological process that is coordinated and controlled by a variety of factors and can be divided into three types: normal abscission caused by senescence or maturation, physiological abscission caused by the plant’s own physiological activities, and stress abscission caused by adversity (Rogers, 2005). During the initiation phase of abscission, the abscission site of the detached organ differentiates into an abscission zone, and the cells in the abscission zone are characterized by a tight arrangement, an accumulation of a number of starch granules, and highly developed intercellular connective filaments compared with the surrounding neighboring cells (Patharkar and Walker, 2018; Yu et al., 2020). Furthermore, during the abscission process, it is often accompanied by an increase in the volume of abscission zone cells, an increase in the size of the cell nucleus both longitudinally and latitudinally with clear nucleolar material, and a noticeable increase in the number of Golgi bodies and mitochondria (Baird et al., 1984).

Plant hormones, as important regulatory signals in the process of abscission, can increase the activity of abscission zone cells and the activity of enzymes related to cell wall metabolism, leading to the degradation of cell wall components, which promotes the abscission of plant organs (Taylor and Whitelaw, 2001). Indole-3-acetic acid (IAA), gibberellins (GAs), and zeatin (ZT) have inhibitory effects on abscission, while abscisic acid (ABA) and ethylene (ETH) accelerate organ abscission (Gulfishan et al., 2019). Generally speaking, high levels of IAA inhibit abscission (Santner and Estelle, 2009). In the “auxin gradient theory”, some scholars suggest that plant organ abscission depends on the ratio of auxin concentration at the distal and proximal ends of the abscission layer (Addicott et al., 1955). Some researchers have suggested that IAA delays abscission by inhibiting the abscission zone’s sensitivity to ETH (Doorn and Stead, 1997). When the auxin polar transport inhibitor of N-(1-Napthyl) phthalamic acid (NPA) was sprayed on apple stalks, the polar transport of the auxin in the abscission zone parts of the stalks was impeded, resulting in the disruption of the dynamic equilibrium between IAA and ETH in the abscission zone, and the process of apple fruit abscission was accelerated (Roberts and Sexton, 1982; Dražeta et al., 2004).

Most studies have shown that high levels of GA_3_ inhibit plant organ abscission (Shi et al., 2023) by promoting auxin biosynthesis and inhibiting the accumulation of ACC (1-Aminocyclopropanecarboxylic Acid) to retard ETH synthesis at the abscission zone (Marciniak et al., 2018). Moreover, GAs can interact with other hormones, thereby participating in the regulation of plant organ abscission (Dong et al., 2021). For example, externally applied GA_3_ significantly promoted the accumulation of endogenous cytokinin (CTK) and IAA, which in turn delayed citrus fruit abscission (Gómez-Cadenas et al., 2000). However, GA_3_ promotes the abscission of grapevine flowers due to GA_3_ weakening the polar transport of IAA (Kühn et al., 2016). Therefore, there is still controversy regarding the role of GA_3_ in the abscission of plant organs. In addition, ZT is a type of CTK and is also involved in the regulation of plant organ abscission. It has been reported that the content of trans-zeatin in the abscission zone of betel nut fruit is significantly lower than that in decalyx fruit (Li et al., 2023). Li (Li, 2016) reported that ZT was closely related to calyx abscission in Korla fragrant pear, and high concentrations of auxin and ZT in the middle and lower parts of the young fruits acted as inhibitors of calyx abscission.

ETH plays a significant role in promoting plant organ abscission (Shi et al., 2023), primarily by promoting the expression of ethylene-responsive factors, which in turn induce transcription and translation of genes related to ethylene-responsive factors, increasing the activity of cell wall hydrolases and leading to the degradation of abscission zone cells, and promoting the occurrence of abscission (Rodrigo and Herrero, 2002). For example, high levels of ethylene in the pedicel of tomatoes directly promote the formation of the abscission zone, accelerating flower abscission (Sundaresan et al., 2018). In addition, ACS (1-aminocyclopropane-1-carboxylic acid synthase) and ACO (1-aminocyclopropane-1-carboxylate oxidase), as key enzymes in ETH biosynthesis encoded by different multigene families, play an important role in the process of abscission (Gamalero et al., 2023). During exogenous ethylene-induced abscission of apple fruits, the expression of the *MdACS5B*, *MdACO1*, and *MdACS5A* genes in the abscission zone significantly increased. Notably, the increased expression of the *MdACO1* gene also led to an increase in ETH content (Cin et al., 2007).

ABA plays a role in positively regulating plant organ abscission (Ito and Nakano, 2015). The increase in ABA content in the lychee fruit abscission zone has been shown to lead to an increase in the expression of the *LcACS1/4/7* and *LcACO2/3* genes mediated by the *LcHB2/3* transcription factor. At this time, the lychee fruit abscission zone was positively regulated by the abscission genes, and this contributed to the decrease in polar transport of IAA, thus further stimulating the production of ETH in the abscission zone of lychee fruit (Zhao et al., 2020). In yellow lupin, ABA treatment significantly increased the transcriptional activities of ACC synthase and ACS, thereby inducing the abscission of yellow lupin flowers (Wilmowicz et al., 2016), further indicating that ABA has a promoting effect on the abscission of plant organs.

Korla fragrant pear (*Pyrus sinkiangensis* Yü.) is originally from the Korla region in Xinjiang. It has a long history of cultivation and is an ancient local variety. Korla fragrant pear is divided into decalyx fruit and persistent calyx fruit. Fruits with a protruding calyx end and persistent sepals are called ‘persistent calyx fruits’. Fruits with a top end that is concave like a funnel and sepals that have fallen off are referred to as “decalyx fruits”. In comparison to decalyx fruits, persistent calyx fruits are generally of lower quality. However, under natural conditions, persistent calyx fruit single plants account for a high proportion, 70%-85%, leading to a decline in the quality of fruits and restricting the sustainable development of the Korla fragrant pear industry. The abscission of calyx in Korla fragrant pear is similar to the abscission of other plant organs, where an abscission zone is formed first, and then abscission occurs (Hao, 2022).

Plant hormones play an important role in regulating the abscission of organs or tissues (Min et al., 2020). At present, studies have been performed on the relationship between the phytohormones and calyx abscission in Korla fragrant pear, but these studies mainly focused on the differences between the phytohormones in decalyx and persistent calyx fruit. More attention has been paid to how plant hormones were distributed in different organs or parts of the same organ during the development of flowers and young fruits, and to their relationship with calyx development. For example, high concentrations of auxin and zeatin in the lower part of the young fruit of Korla fragrant pear inhibited calyx abscission, while low concentrations of IAA, ZT, and high concentrations of ABA promote calyx abscission (Li, 2016). Existing hormone measurement methods such as gas chromatography, liquid chromatography, and mass spectrometry can well interpret the changes in plant hormone levels during organ abscission. However, the results obtained are actually the average hormone levels of hundreds or even thousands of cells, and they cannot provide precise localization analysis of hormones. Moreover, due to the significant differences in cells within and around the abscission zone during the calyx abscission process, the original changes in hormones in the abscission zone often only occur in a single or a few tens of cells in the abscission zone. Therefore, understanding the spatial distribution of hormones is very important (Li and Liu, 2016; Ma et al., 2019). MALDI-MSI (Matrix-Assisted Laser Desorption Ionization-Mass Spectrometry Imaging) technology is a novel molecular imaging technique that has overcome the bottlenecks of traditional hormone detection methods that lose spatial information (Alexandrov, 2020; Taylor et al., 2021) and is capable of determining the spatial distribution, relative content, and structural information of various molecules within the abscission zone tissues in three dimensions. The qualitative and quantitative analysis of plant hormone molecules in the abscission zones of decalyx fruits and the corresponding abscission zones of persistent calyx fruits of Korla fragrant pear can be achieved by using MALDI-MSI technology, which allowed for the visualization of the spatial location of hormones, thereby better elucidating the regulatory role of plant hormones on the abscission of the calyx. Therefore, the objectives of this study were as follows: (1) to clarify the characteristics of calyx abscission and the developmental process of the abscission zone; (2) to elucidate the changes in plant hormone content and the spatial distribution within the abscission zone during the process of calyx abscission. Our research findings have enriched our understanding of the regulatory mechanism of plant hormones on the abscission of the calyx, and provide a theoretical basis for the study of “exogenous plant growth regulators for the regulation the calyx abscission”, which is conducive to increasing the calyx abscission rate and improving fruit quality.

## 2 Materials and methods

### 2.1 Description of the experimental site and plant material

The experimental site was located at the experimental base of the Korla Fragrant Pear Research Centre, Bayingolin Mongolian Autonomous Prefecture, Xinjiang (86°00′39.09″E, 41°36′53.86″N), with an average annual temperature of 11.4°C, an average annual sunshine exposure of 2986 h, and an average annual precipitation of 58.6 mm. The plant material was the Korla fragrant pear 6a cultivar, with a plant spacing of 2 m×4 m. The trees had a sparse, layered structure, and uniform growth potential. The sparse, layered structure has a distinct main trunk, with the main branches arranged in layers along the central trunk. The first layer is composed of three closely spaced main branches. The second layer is composed of 2-3 main branches. Each main branch bears 2-3 lateral branches.

### 2.2 Sampling methods

**Figure 1:**
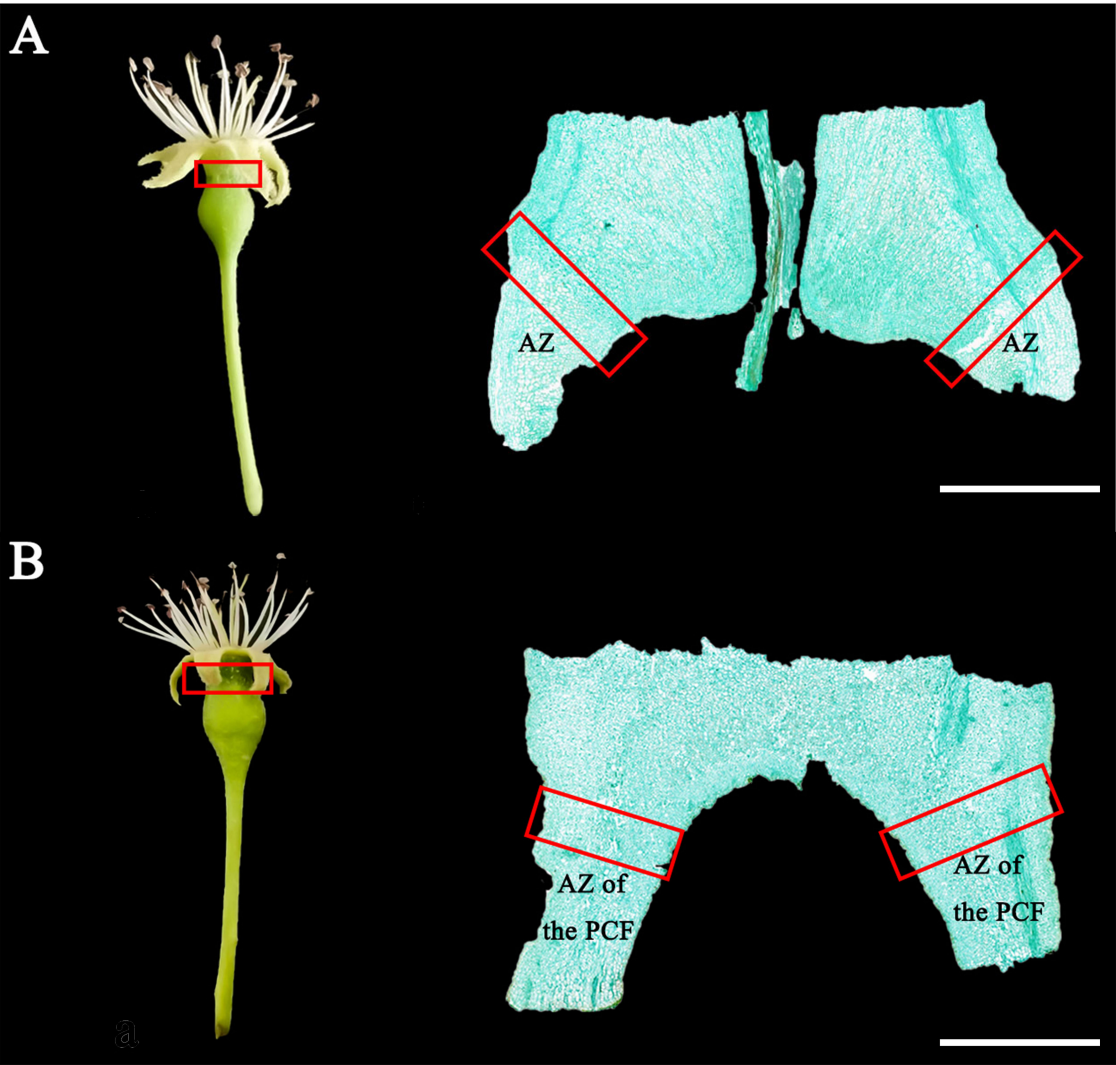
Morphology of young fruits of Korla fragrant pear and the anatomical structure of the abscission zone of decalyx fruits and the corresponding part of the abscission zone of persistent calyx fruits during the abscission zone formation period. **(A)** is the decalyx fruit; **(B)** is the persistent calyx fruit. Staining was done with Safranin-Fast Green. AZ, Abscission zone of the decalyx fruit; AZ of the PCF, Corresponding part of the abscission zone of the persistent calyx fruit. Bars = 2000 μm.

The process of calyx abscission in Korla fragrant pear can be divided into three periods as follows: (1) before, (2) during, and (3) after the formation of the abscission zone. Previous studies have shown that as the floral position ascends in Korla fragrant pear inflorescences, the ability of the calyx to abscise increases, and the capacity for calyx persistence weakens. Consequently, in an inflorescence, the first and second-order floral positions usually produce persistent calyx fruits, while the fourth and fifth-order floral positions typically yield decalyx fruits (He et al., 2007). Therefore, in the early stages of calyx abscission when the abscission of the calyx could not be observed from the appearance, young fruits from the first and second-order positions serve as samples of persistent calyx fruits, while young fruits from the fourth and fifth-order positions act as samples of decalyx fruits. Young persistent calyx fruits and decalyx fruits were randomly selected from the middle and lower parts of the periphery canopy during the three periods mentioned above (**Figure 1**). During the paraffin sectioning process, the staining method refers to Yu’s method (Yu et al., 2020). For the sample preparation, a scalpel was used to sever the calyx tube at its base where it was attached to the fruit, followed by the manual removal of the petals and calyx. In the case of decalyx fruits, the tissue in the abscission zone, encompassing the calyx tube, several layers of distal cells, and the adjacent cells along the separation line, was carefully extracted. For the persistent calyx fruits, the equivalent area in the distal and proximal region of the calyx was similarly extracted. The calyx part of the young fruit was fixed with glutaraldehyde, sectioned into 1mm³ small tissue blocks, and placed under an electron microscope to observe the cellular ultrastructure.

### 2.3 Investigation of the Korla fragrant pear calyx abscission patterns

#### 2.3.1 Observations of the external characteristics during calyx abscission

After the pollination of the Korla fragrant pear was completed, observations were conducted of the external morphological characteristics of the calyx abscission zone during the calyx abscission process (before, during, and after abscission zone formation).

#### 2.3.2 Cellular ultrastructure observation of the cells in the abscission zone during the Korla fragrant pear calyx abscission process

According to Bar’s method (Bar et al., 2011), with some modifications, the sample was fixed with a 1% osmic acid fixative at room temperature for 7 h away from light and was rinsed with 0.1M phosphate buffer PB (pH=7.4) 3 times, 15 min each time. The samples were dehydrated with 30%-50%-70%-80%-95%-100%-100% alcohol successively for 1 h each time. Anhydrous ethanol: acetone=3:1, 0.5h, anhydrous ethanol: acetone=1:1, 0.5h, anhydrous ethanol: acetone=1:3, 0.5h. After the dehydration was complete, the sample was placed in an oven at 37°C overnight, positioned behind the embedded plate. It was then polymerized in an oven at 60°C for 48 h, and the resin block was removed after completion. The resin blocks were cut into 60-80 nm ultra-thin sections by an ultra-thin microtome and placed in 150 mesh cupola film copper mesh. The dye was stained with a 2% uranium acetate-saturated alcohol solution for 8 min. The samples were washed with 70% alcohol three times and then cleaned with ultra-pure water three times. Subsequently, they were stained with a 2.6% lead citrate solution for 8 minutes, taking care to avoid exposure to light. After staining, the material was rinsed again with ultra-pure water three times and briefly blotted with filter paper. The copper mesh slices were then placed into a copper mesh box and left to dry overnight at room temperature. Finally, the ultrastructure of the isolated cells was observed using an HT7800/HT7700 transmission electron microscope, and the resulting images were collected and analyzed.

### 2.4 Determination of plant hormone content

IAA, ABA, GA_3_, and ZT were determined by high-performance liquid chromatography (HPLC) according to the assay method of Qi et al (Qi et al., 2021). ETH was determined by high-performance gas chromatography (GC), and the method of ETH determination was modified according to Rodriguez et al (Rodriguez et al., 2010). The standard substance information is shown in **Supplementary Table S1** in the **Supplementary Material**. Each hormone was measured with three biological replicates. Five grams of sample was weighed into a glass vial, corked with a rubber stopper, and the slit was sealed with a sealing film and stored at room temperature (20°C) for 24 h. The glass container was gently shaken to completely remove the air inside the syringe, and the airtight needle stem was pushed and pulled repeatedly four times. A volume of 30 mL was collected from the glass container using the drainage method and injected into the gas chromatograph for detection.

### 2.5 Detection of the spatial distribution of plant hormones

#### 2.5.1 Sample Preparation

The tissue was frozen and sliced using the Leica CM1950 cryotome, which was fixed to the sample holder to adjust the angle and orientation of the sample, and the sample was sliced using the microtome, after which the slices were transferred to the pre-cooled ITO slide. Finally, the ITO slides containing tissue slices were dried for 30 minutes with a vacuum drying instrument (**Supplementary Table S2** in the **Supplementary Material**).

#### 2.5.2 Mass spectrometry imaging and data processing

Substrate spraying: A 15 mg/mL solution of DHB (2,5-dihydroxybenzoic acid) was prepared using a 90:10 (v/v) acetonitrile to water solution. This DHB matrix solution was evenly applied to ITO slides with tissue sections using a TM-Sprayer matrix sprayer. The instrumental settings were: temperature at 60°C, flow rate at 0.12 mL/min, pressure at 6 psi, with 26 cycles of gas application, and a 5-second drying interval between each cycle.

**Figure 2:**
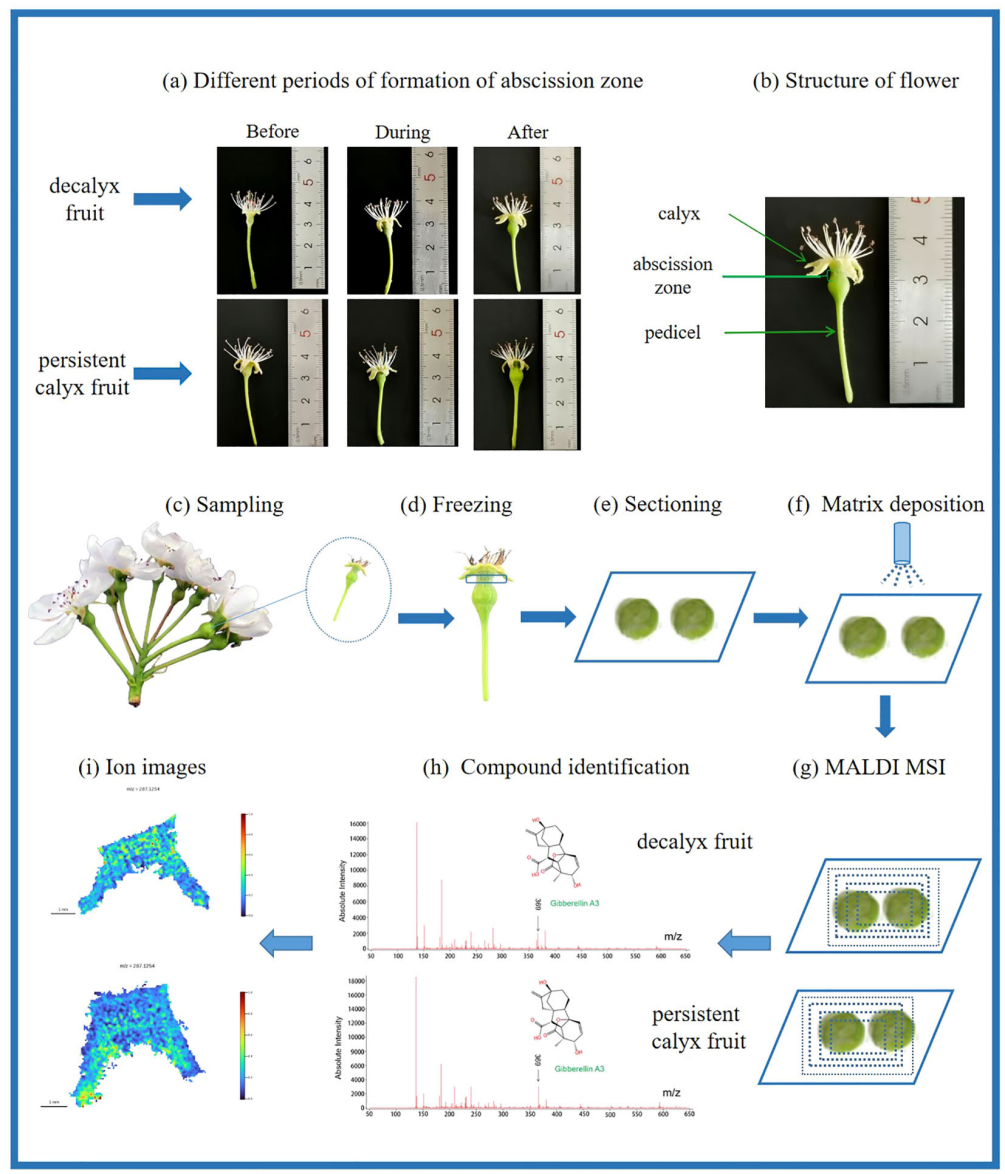
The process of sample preparation for mass spectrometry imaging of Korla fragrant pear calyx. **(A)** Developmental periods of abscission zone formation. The samples were thawed, cut into 40 μm sections, and mounted on ITO-laminated slides; **(B)** Structure of flower; **(C)** Sample collection; **(D)** Frozen fresh samples; **(E)** Frozen sections; **(F)** DHB matrix spraying; **(G)** MALDI-MSI data acquisition; **(H)** Compound identification; **(I)** MALDI-MSI image exportation.

The ITO slide was placed on the target plate of the mass spectrometer, Datalmaging (Bruker) software was selected to outline the tissue area, and the imaging resolution was set to 50 μm (i.e., the minimum cell size of the two-dimensional dot matrix was 50 μm × 50 μm). The imaging region was divided into a two-dimensional grid composed of 20,000 and 25,000 points according to the size, the imaging range was set to 50-650 Da, the tissue samples were detected under the same laser energy, the laser beam irradiated the tissue area on the target plate through the grating, and the samples were continuously scanned. The tissue samples were ionized and resolved with the matrix under the excitation of the laser beam, and the released molecules were identified by the mass spectrometer to obtain the mass-to-charge ratio (m/z) information and the raw peak intensity data of each pixel of the samples (raw data). The raw data were imported into SCiLS Lab software for smoothing and root mean square (RMS) standardization, and the relative intensities of different m/z at each spatial point were obtained and converted into pixels on the imaging thermogram (**Figure 2**). Additionally, the target peaks of the target substances (MS first-level information) were subjected to *in situ* secondary fragmentation on the tissue to obtain the secondary mass spectra (MS/MS second-level fragment ion information) collected from the tissue. By comparing these spectra with the secondary mass spectra of plant hormone standards, the identification of the substances can be performed more accurately. (**Supplementary Figures S1-S5** in the **Supplementary Material**).

### 2.6 Data Processing and Statistical analysis

Statistical Product and Service Solutions (SPSS) 25.0 software was used for the statistical analysis of the data, and a *t-test* was used to determine the significance of the differences (*P*<0.05). Microsoft Excel 2019 and Origin 2018 were used for graphing, and Adobe Photoshop CC 2019 was used to integrate the images.

## 3 Results

### 3.1 External morphological changes during the calyx abscission process in Korla fragrant pear

**Figure 3:**
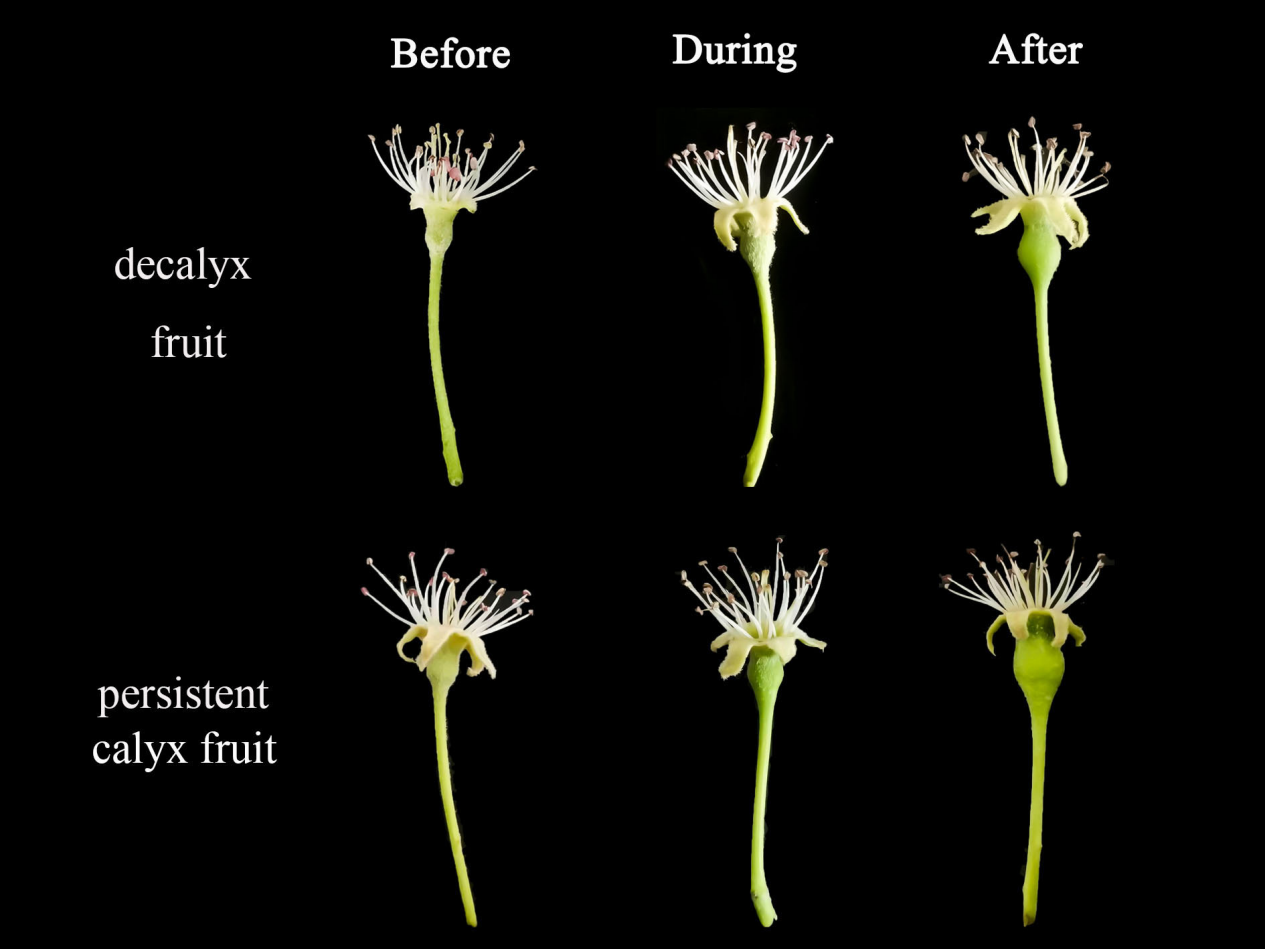
External morphological changes during the calyx abscission process of Korla fragrant pear.

The external morphological characteristics of the Korla fragrant pear showed evident differences between the decalyx fruit and persistent calyx fruit during the various stages of abscission zone formation (**Figure 3**). Before the formation of the abscission zone, for decalyx fruit, the calyx and stamen gynoecium development were normal, the surface of the young fruit had epidermal hairs, and the fruit size was small. The external characteristics of the persistent calyx fruit were similar to that of the decalyx fruit, with no significant differences. During abscission zone formation, for the decalyx fruit, the stamen and pistils began to turn brown compared with that before zone formation, the calyx and fruit continued to grow normally, and a relatively indistinct ring-like abscission zone could be observed at the junction between the fruit and calyx tube. The trichomes on the surface of the young fruits began to decrease, and the fruit volume gradually increased, while the persistent calyx fruit stamens anthers began to turn brown, the calyx grew normally, the trichomes on the surface of the young fruit began to decrease, and its volume gradually increased. After the formation of the abscission zone, the stamens and pistils of the decalyx fruit clearly turned brown, some of the stamens became black-brown, and the calyx still grew normally. Notably, at the intersection of the calyx tube and the fruit, an evident abscission zone could be observed on a yellow ring. Thus, the detachment of calyces could be accurately determined. The calyx of the persistent calyx fruit developed normally. Unlike the decalyx fruit, the calyx tube of the persistent calyx fruit elongated without forming a yellow ring at the junction with the fruit, which grew and developed to become a part of the fruit’s apex, accompanying the overall growth and development of the fruit.

### 3.2 Cellular ultrastructure observation during the calyx abscission process in Korla fragrant pear

**Figure 4:**
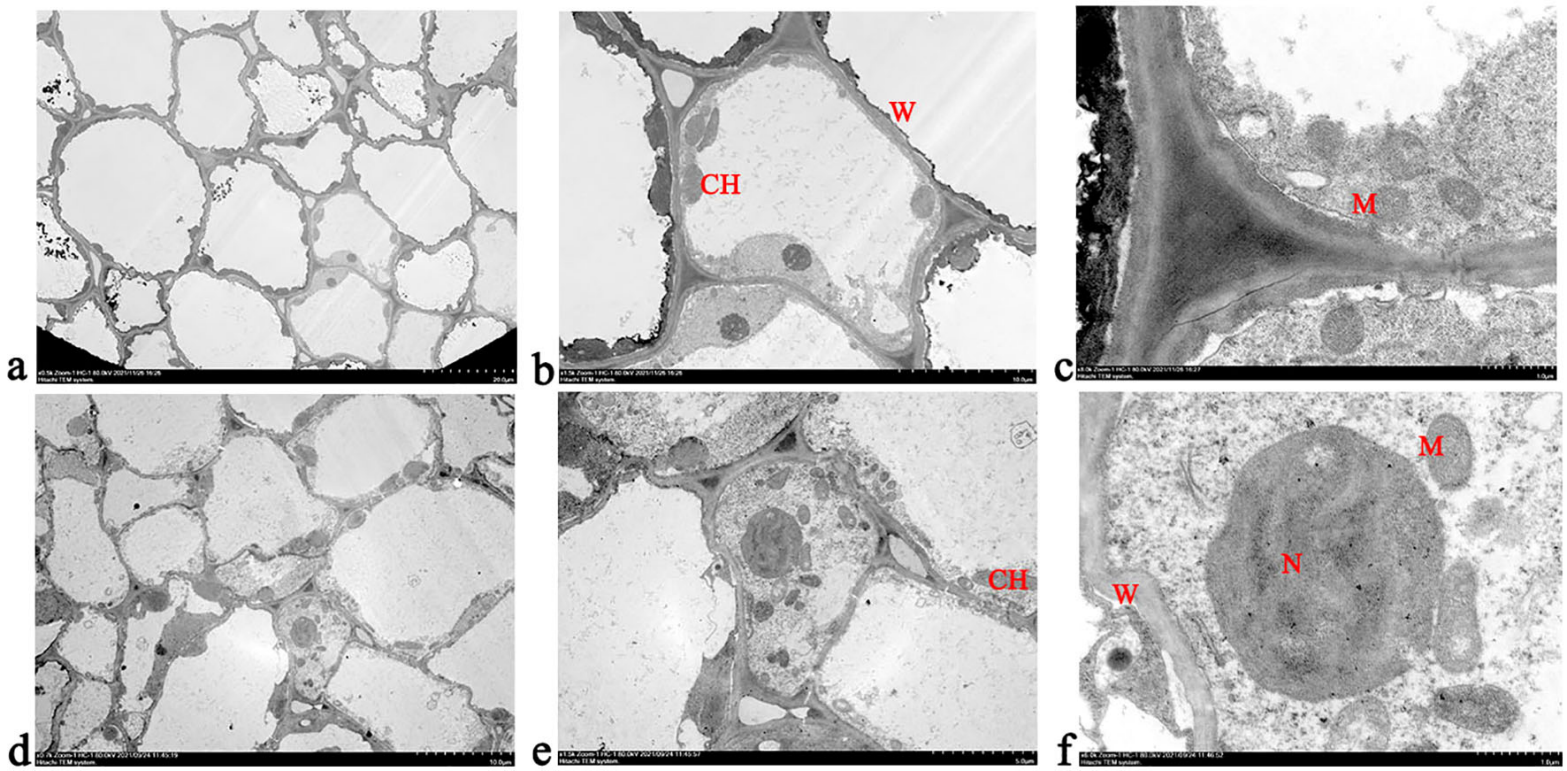
Cellular ultrastructure observation before the formation of the abscission zone in Korla fragrant pear. **(A–C)** represent the ultrastructure of the decalyx fruit under different magnifications in various fields of view (**A**, 20 μm; **B**, 5.0 μm; **C**, 1.0 μm); **(D–F)** represent the ultrastructure of the persistent calyx fruit under different magnifications in various fields of view (**D**, 20 μm; **E**, 5.0 μm; **F**, 1.0 μm). CH, chloroplast; M, mitochondrion. N, nucleus; W, cell wall.

A significant difference was observed in the cellular ultrastructure between the abscission zone of the decalyx fruit and the corresponding parts of the abscission zone of the persistent calyx fruit (**Figure 4**). Specifically, before the formation of the abscission zone, the cells in the abscission zone of the decalyx fruit were not fully developed, the cell walls were weak, a small number of mitochondria were located around the cell wall, and the chloroplasts were not yet mature (**Figures 4A–C**). However, the cells in the corresponding parts of the abscission zone of the persistent calyx fruit had weak cell walls and large nuclei, mitochondria were irregularly distributed in the cells, and the chloroplasts were in the early stage of development with a long ellipsoid shape (**Figures 4D–F**).

**Figure 5:**
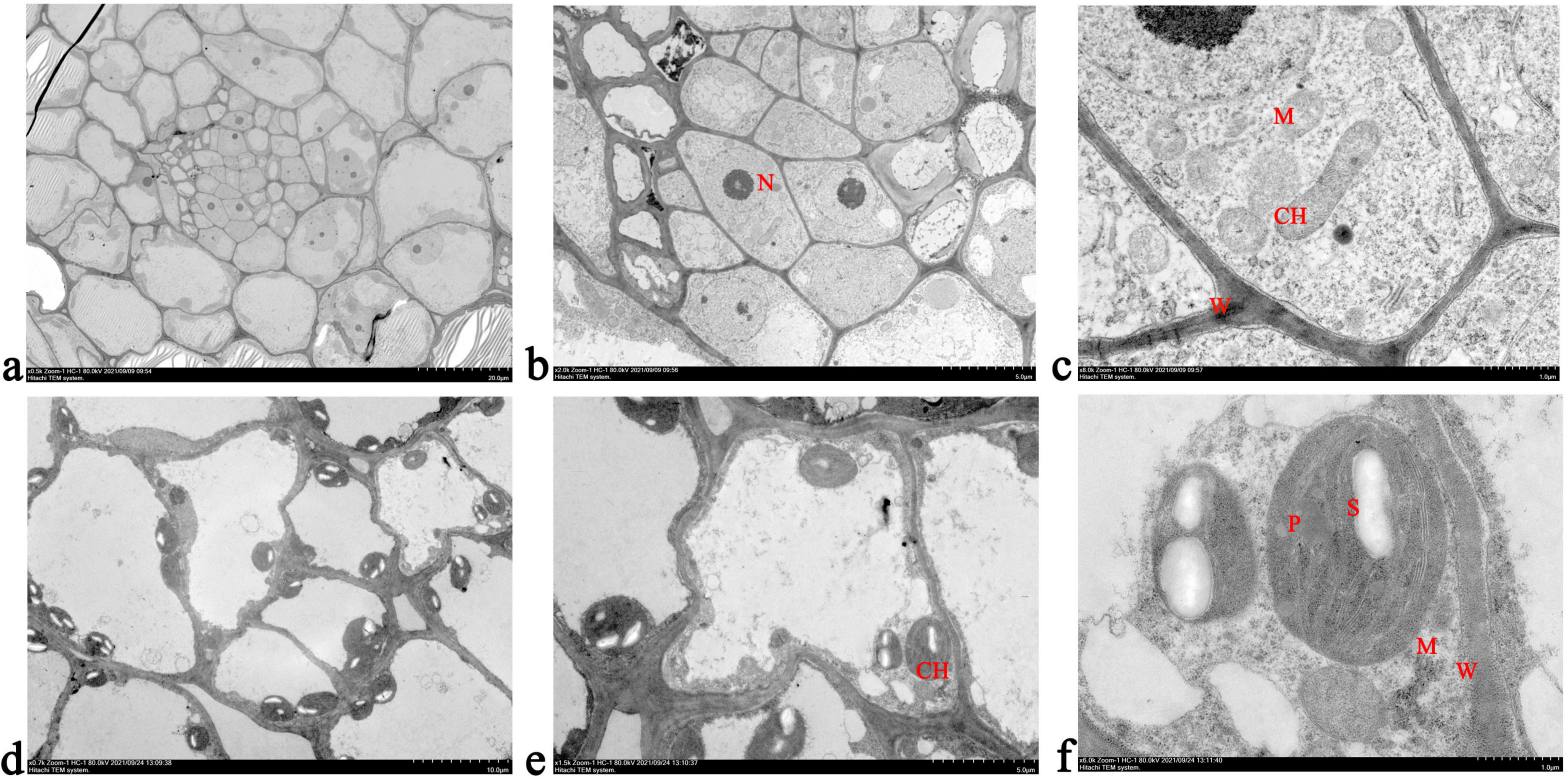
Cellular ultrastructure observation during the formation of the abscission zone in Korla fragrant pear. **(A–C)** represent the ultrastructure of the decalyx fruit under different magnifications in various fields of view (**A**, 20 μm; **B**, 5.0 μm; **C**, 1.0 μm); **(D–F)** represent the ultrastructure of the persistent calyx fruit under different magnifications in various fields of view (**D**, 20 μm; **E**, 5.0 μm; **F**, 1.0 μm). CH, chloroplast; M, mitochondrion; N, nucleus; P, plastoglobule; S, starch grain.

During abscission zone formation, the cells in the abscission zone of the decalyx fruit were slightly edematous, with intact cell membranes and continuous cell walls of uniform thickness, and no evident plasmolysis was observed. The nuclei were oval in shape, with the nuclear membranes becoming fuzzy, a moderate number of chloroplasts, an intact membrane structure, and a uniform stroma, and the number of mitochondria is adequate, with slight swelling (**Figures 5A–C**). The cell structure in the persistent calyx fruit was found to be normal. The number of chloroplasts was high, and more starch grains appeared inside the chloroplasts. The membrane structure was intact, the number of plastoglobules was abundant, the number of mitochondria was adequate, with slight swelling, and the morphology was normal (**Figures 5D–F**).

**Figure 6:**
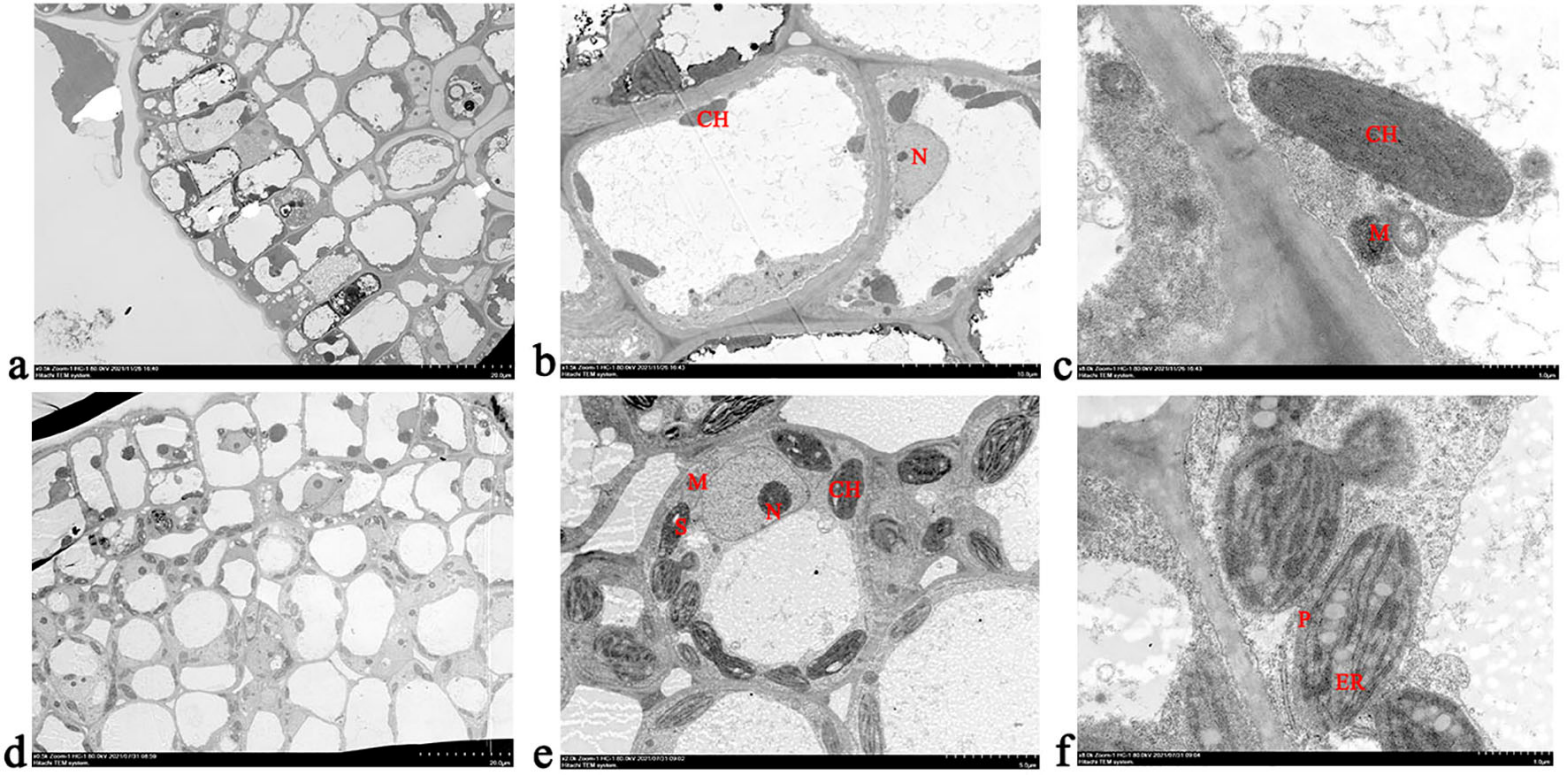
Cellular ultrastructure observation after the formation of the abscission zone of Korla fragrant pear. **(A–C)** represent the ultrastructure of the decalyx fruit under different magnifications in various fields of view (**A**: 20 μm; **B**: 5.0 μm; **C**: 1.0 μm); **(D–F)** represent the ultrastructure of the persistent calyx fruit under different magnifications in various fields of view (**D**: 20 μm; **E**: 5.0 μm; **F**: 1.0 μm). CH, chloroplast; ER, endoplasmic reticulum; M, mitochondrion; N, nucleus; P, plastoglobule; S, starch grain.

After the formation of the abscission zone, the protective layer outside the cell wall at the abscission zone of the decalyx fruit had formed, and a deformation of the structure of the outer cell wall occurred, and, with an increase in vesicle numbers and size, a blurring of the boundary of the vesicle membrane, and a reduction in the cell nucleus size (**Figures 6A–C**). In contrast, the cell walls of the persistent calyx fruit cells gradually thickened, the number of mitochondria was moderate, the chloroplast structure was well developed and increased in number, the number of plastoglobules was abundant, the stroma was homogeneous, the endoplasmic reticulum was formed, and distinct starch grains filled the chloroplasts (**Figures 6D–F**).

### 3.3 Visualization of the plant hormones during calyx abscission in Korla fragrant pear

#### 3.3.1 Ion intensity analysis of plant hormones

IAA, GA_3_, ZT, ABA, and ETH, as the five common major phytohormones, play an essential role in the process of calyx abscission in Korla fragrant pear and were visualized using MALDI-MSI in this study. It was found that the ion intensities of IAA (m/z 193.0974), GA_3_ (m/z 369.1258), ZT (m/z 352.1519), and ABA (m/z 287.1254) were relatively stable in the abscission zone of both the decalyx fruit and the corresponding part of the persistent calyx fruit (**Supplementary Figure S6** in the **Supplementary Material**). As ETH is a gaseous molecule with a relatively small molecular weight and an unstable existence, the ion signal intensity of ETH has not been detected by MALDI-MSI to date. However, the long-distance transport of ETH relies on the transport of its immediate precursor, ACC, in the xylem, and using MALDI-MSI, the spatial distribution of ACC (m/z 124.0386) could be clearly observed. The molecular weight of ACC was relatively stable, and the relative quantitative results of MALDI-MSI were detected. Therefore, this study used MALDI-MSI to detect the spatial distribution of ACC in the abscission zone and, in combination with gas chromatography, clarified the mechanism by which ETH played a role in the abscission zone process in Korla fragrant pear. The biosynthetic pathway of ETH is shown in **Supplementary Figure S7** in the **Supplementary Material** (Satoh and Yang, 1991).

#### 3.3.2 Visualization analysis of IAA

**Figure 7:**
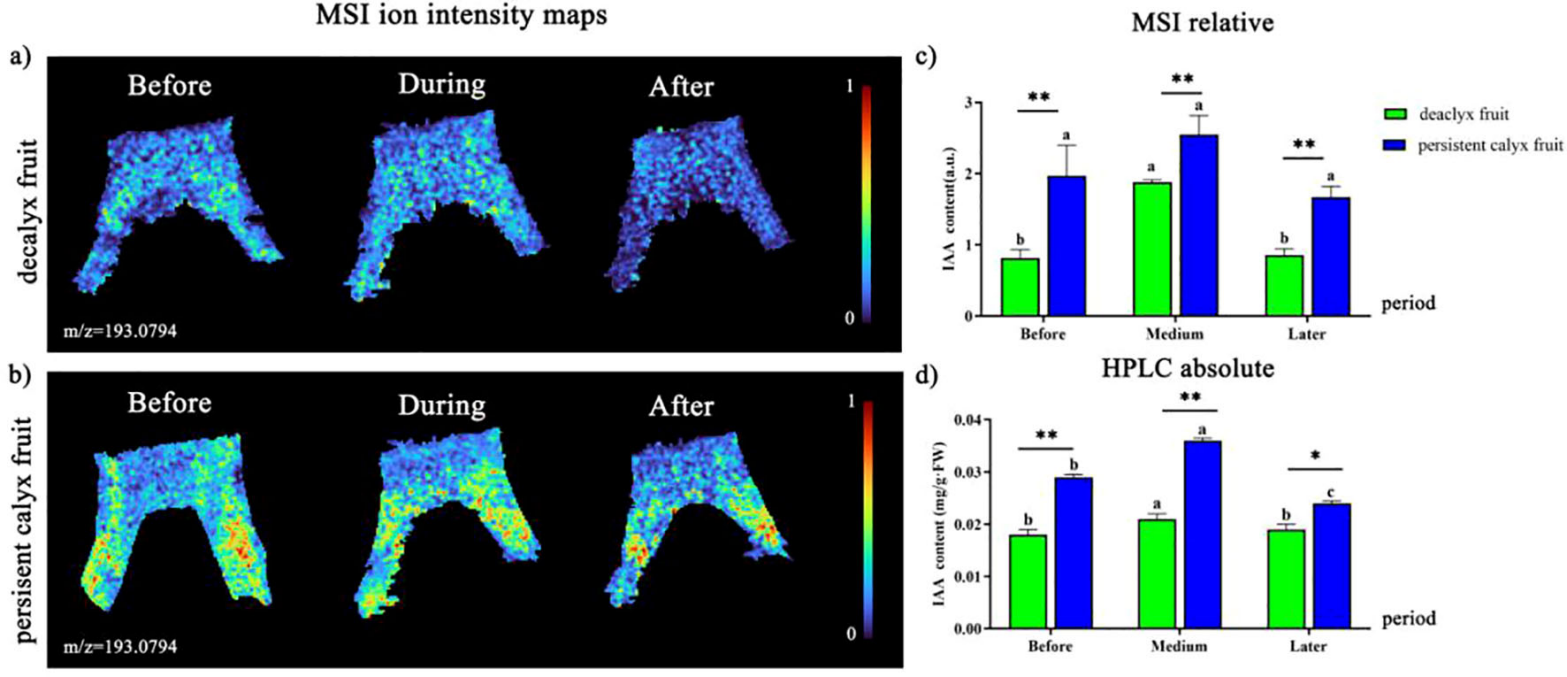
Visualized analysis of IAA during the calyx abscission process of Korla fragrant pear by MALDI-MSI and HPLC. **(A)** Mass spectrometry imaging of IAA ([M+NH]^4+^, m/z 193.0974 m/z) in the abscission zone of the decalyx fruit calyx, and **(B)** mass spectrometry imaging of IAA ([M+NH]^4+^, m/z 193.0974 m/z) in the corresponding abscission zone area of the persistent calyx fruit. **(C)** HPLC determination of the IAA content. **(D)** MALDI-MSI determination of IAA. A *t-test* was used to analyze the difference in phytohormone content in the abscission zone of the decalyx fruits and the corresponding part of the abscission zone of the persistent calyx fruits. *: Significant; **: Highly significant.

A significant difference in the distribution of IAA in the calyx abscission zone of the decalyx fruits and the corresponding part of the persistent calyx fruits was observed (**Figure 7**). Thus, the brighter the color, the stronger the signal intensity of the IAA ion. In the decalyx fruits, IAA ([M+NH]^4+^, m/z 193.0974) was more uniformly distributed in the abscission zone cells and the adjacent cells along the proximal side of the entire calyx tube and the separation line, with no significant differences (**Figure 7A**). In contrast, the distribution of IAA in the calyx tube of persistent calyx fruit was closer to the calyx site than to the site of attachment of the young fruit during the whole calyx abscission process. Moreover, the color on one side of the calyx was brighter than where it connected to the calyx tube, indicating that IAA was not uniformly distributed in the calyx tube area of the persistent calyx fruit, but was enriched in certain areas (**Figure 7B**). In addition, from the results of the relative quantification of MALDI-MSI (**Figure 7C**), in the persistent calyx fruits, the IAA content within the corresponding part of the calyx abscission zone was significantly higher before (1.97), during (2.55), and after (1.67) the formation of the abscission zone compared to the decalyx fruits. Moreover, the IAA content in both the decalyx fruit (1.88) and persistent calyx fruit (2.55) peaked during the abscission zone formation period. Moreover, the HPLC quantitative results were consistent with the relative quantitative results of the MALDI-MSI analysis, indicating that during the abscission zone formation period, the IAA content in the calyx abscission zone of the decalyx fruits and the persistent calyx fruits was significantly higher by 14.2% and 19.4%, respectively, before the abscission zone formation, and by 9.5% and 33.3%, respectively, after abscission zone formation, compared to before abscission zone formation. Additionally, the IAA content in the persistent calyx fruits (0.029 mg/g·FW during the pre-formation period, 0.036 mg/g·FW during the calyx abscission, and 0.024 mg/g·FW after the abscission zone formation) were all significantly higher than those in the decalyx fruits (0.018 mg/g·FW, 0.021 mg/g·FW, and 0.019 mg/g·FW, respectively). The IAA content of the persistent calyx fruit was 37.9%, 41.7%, and 20.8% higher than that of decalyx fruits before, during, and after the formation of the abscission zone, respectively (**Figure 7D**).

#### 3.3.3 Visualization analysis of GA_3_

**Figure 8:**
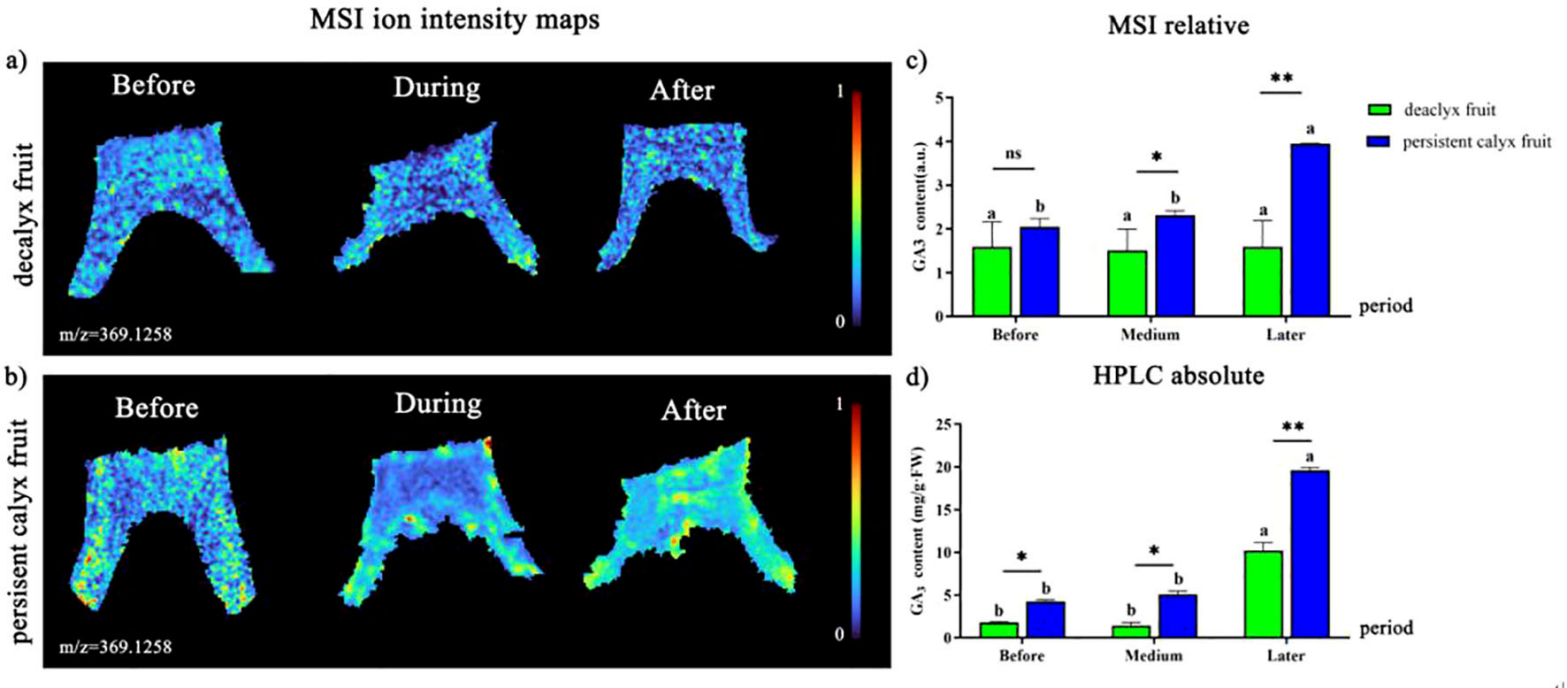
Visualized analysis of GA_3_ during the calyx abscission process of Korla fragrant pear by MALDI-MSI and HPLC. **(A)** Mass spectrometry imaging of GA_3_ ([M+Na]^+^, m/z 369.1258 m/z) in the abscission zone of the decalyx fruit calyx, and **(B)** mass spectrometry imaging of GA_3_ ([M+Na]^+^, m/z 369.12584 m/z) in the corresponding abscission zone area of the persistent calyx fruit. **(C)** HPLC determination of the GA_3_ content. **(D)** MALDI-MSI determination of GA_3._ A *t-test* was used to analyze the difference in phytohormone content in the abscission zone of the decalyx fruits and the corresponding part of the abscission zone of the persistent calyx fruits. *: Significant; **: Highly significant. "ns" stands for "non-significant".

A brighter color indicated a stronger ionic signal intensity of the GA_3_, and the following results were shown. In the decalyx fruits, after the formation of the abscission zone, the distribution of GA_3_ in the abscission zone of the decalyx fruits was more uniform, and some GA_3_ ([M+Na]^+^, m/z 369.1258) ions were mainly concentrated at the connection between the calyx tube and the young fruit (**Figure 8A**). However, in the persistent calyx fruits, the ionic signal intensity of GA_3_ was unevenly distributed in the calyx tube region and its related areas before and during the formation of the abscission zone and was mainly concentrated on the side of the calyx tube close to the calyx rather than the connection to the young fruit (**Figure 8B**). In contrast, the distribution of GA_3_ in the decalyx fruit was more uniform throughout the calyx tube and the proximal side of the abscission zone cells and neighboring cells, with no significant differences. In addition, from the results of relative quantification of MALDI-MSI (**Figure 8C**), the GA_3_ content in the corresponding part of the abscission zone of the persistent calyx fruit was significantly greater than that in the decalyx fruits before (2.05), during (2.32) and after (3.95) the formation of the abscission zone. Moreover, the content reached a maximum after the formation of the abscission zone (3.95). In addition, the HPLC quantitative results were consistent with the relative quantitative analysis of MALDI-MSI results, indicating that after the formation of the abscission zone, the GA_3_ content in the abscission zone of the decalyx fruit calyx and the corresponding part of abscission zone of the persistent calyx fruit were significantly higher than those before the formation by 82.3% and 78.2% respectively, and during the abscission zone formation period by 85.9% and 74.0% respectively. Additionally, the GA_3_ content of the corresponding part of the abscission zone of the persistent calyx fruit was significantly greater than that in the decalyx fruit, with levels of 4.26 mg/g·FW before the formation of the abscission zone, 5.09 mg/g·FW during the abscission zone formation period, and 19.61 mg/g·FW after the abscission zone formation period. Moreover, these values were significantly greater than those of the decalyx fruit (1.80 mg/g·FW, 1.44 mg/g·FW, and 10.22 mg/g·FW), The GA_3_ content of the persistent calyx fruit was 57.7%, 71.7%, and 47.8% higher than that of the decalyx fruits before, during, and after the formation of the abscission zone, respectively (**Figure 8D**).

#### 3.3.4 Visualization analysis of ZT

**Figure 9:**
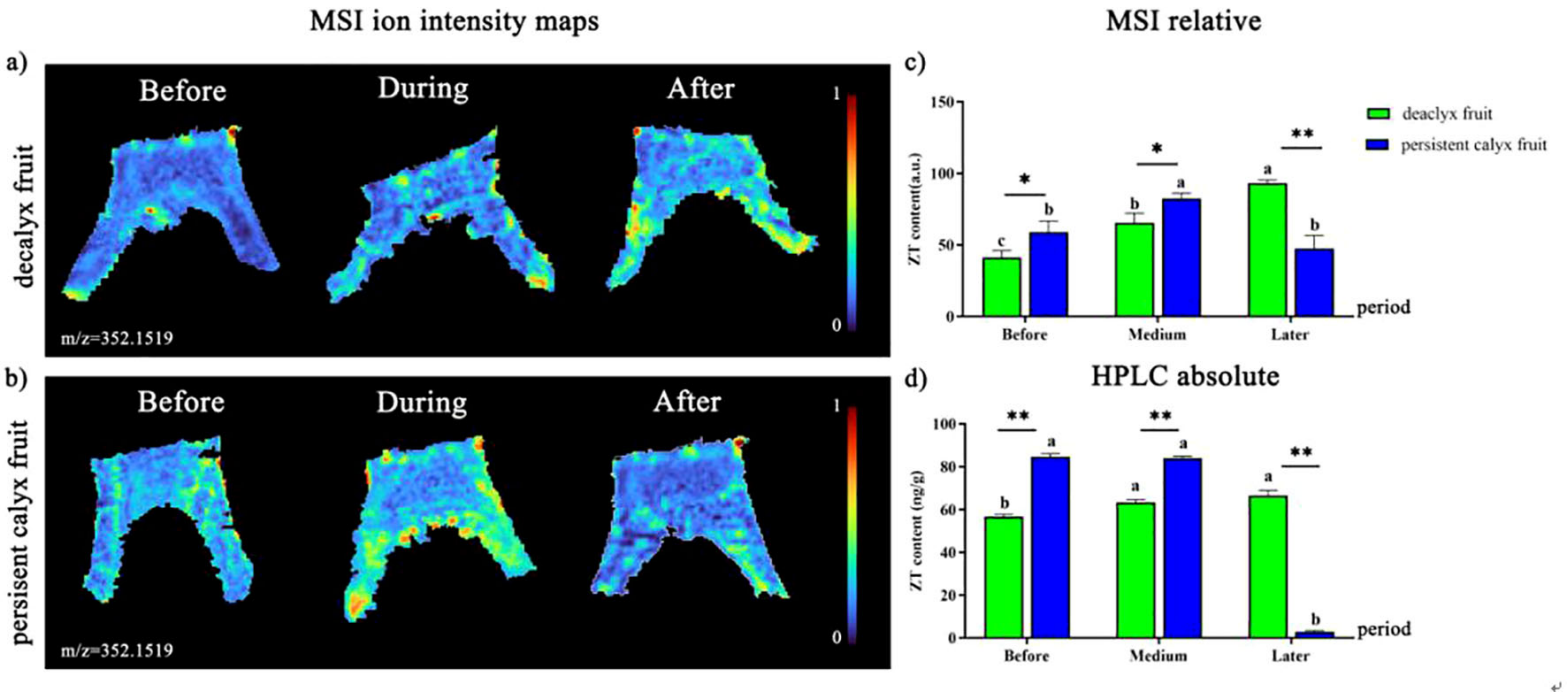
Visualized analysis of ZT during the calyx abscission process of Korla fragrant pear by MALDI-MSI and HPLC. **(A)** Mass spectrometry imaging of ZT ([M+H]^+^, m/z 352.1519 m/z) in the abscission zone of the decalyx fruit calyx, and **(B)** mass spectrometry imaging of ZT ([M+H]^+^, m/z 352.1519 m/z) in the corresponding abscission zone area of the persistent calyx fruit. **(C)** HPLC determination of the ZT content. **(D)** MALDI-MSI determination of ZT. A *t-test* was used to analyze the difference in phytohormone content in the abscission zone of the decalyx fruits and the corresponding part of the abscission zone of the persistent calyx fruits. *: Significant; **: Highly significant.

The brighter color indicated that the ionic signal intensity of ZT was stronger. Specifically, the ionic signal intensity of ZT ([M+H]^+^, m/z 352.1519) in the decalyx fruits was distributed more uniformly before and during abscission zone formation, but after the abscission zone had formed, it became partially concentrated in the abscission zone cells and adjacent cells on the proximal side of the calyx tube and separation line (**Figure 9A**). However, the ionic signal intensity of ZT was distributed more uniformly in the calyx tube of the persistent calyx fruits and their related areas before and after abscission zone formation, and during the abscission zone formation period, the overall distribution of the ion signal intensity of ZT was uneven, and it was mainly concentrated on the side and edge areas close to the calyx (**Figure 9B**). Moreover, from the results of relative quantification of MALDI-MSI (**Figure 9C**), before (58.89) and during the formation of the abscission zone (82.50), the ZT content at the corresponding part of the abscission zone in the calyx of the persistent calyx fruit was significantly higher than that in the decalyx fruit (41.24, 65.51), and the relative ZT content reached a maximum during abscission zone formation (82.50). However, the ZT content in the abscission zone of the decalyx fruits was significantly greater than that in the persistent calyx fruits after the formation of the abscission zone, with a content of 93.39, compared to 47.45 in the persistent calyx fruit. In addition, the HPLC quantification results were consistent with the MALDI-MSI relative quantification results. Specifically, the ZT content was significantly greater in the corresponding parts of the calyx abscission zone in the persistent calyx fruit before (84.66 ng/g·FW) and during the abscission zone formation period (84.03 ng/g·FW) than that in the decalyx fruit, which was 56.71 ng/g·FW before the formation and 63.35 ng/g during the formation of abscission zone. However, after the formation of the abscission zone, the ZT content in the decalyx fruit (66.45 ng/g·FW) was significantly higher than that in the persistent calyx fruit (2.87 ng/g·FW), and reached a peak at this time. The content of ZT before and during the formation of the abscission zone in the corresponding parts of the abscission zone of the persistent calyx fruit was significantly higher than that of the decalyx fruits by 33.0% and 24.6% (**Figure 9D**).

#### 3.3.5 Visualization analysis of ABA

**Figure 10:**
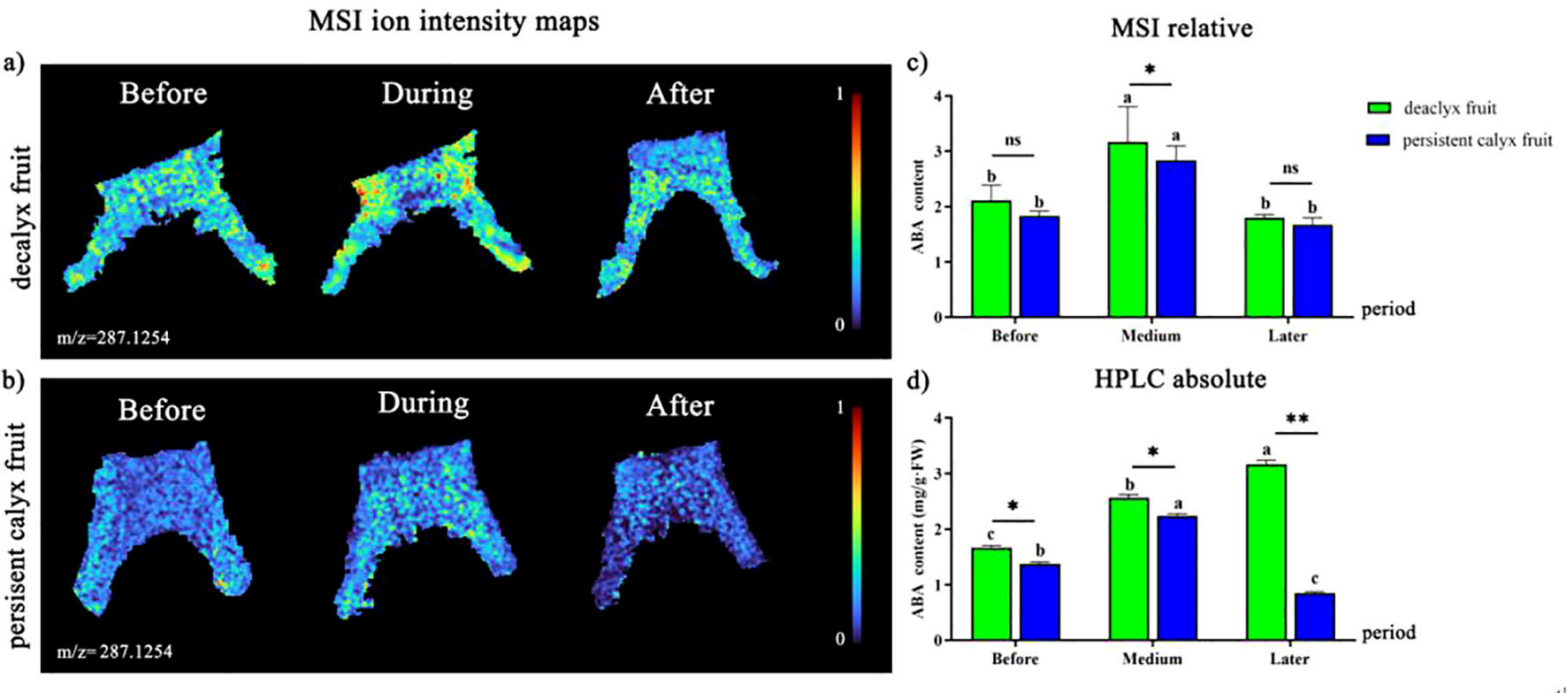
Visualized analysis of ABA during the calyx abscission process of Korla fragrant pear by MALDI-MSI and HPLC. **(A)** Mass spectrometry imaging of ABA ([M+Na]^+^, m/z 287.1254 m/z) in the abscission zone of the decalyx fruit calyx, and **(B)** mass spectrometry imaging of ABA ([M+Na]^+^, m/z 287.1254 m/z) in the corresponding abscission zone area of the persistent calyx fruit. **(C)** HPLC determination of the ABA content. **(D)** MALDI-MSI determination of ABA. A *t-test* was used to analyze the difference in phytohormone content in the abscission zone of the decalyx fruits and the corresponding part of the abscission zone of the persistent calyx fruits. *: Significant; **: Highly significant. "ns" stands for "non-significant".

The brighter color indicated a stronger ionic signal intensity of ABA. The ionic signal intensity of ABA ([M+Na]^+^, m/z 287.1254) before the abscission zone formation in the decalyx fruits was distributed more uniformly in the calyx tube and its related regions, which was generally inhomogeneous during and after the formation of the abscission zone (**Figure 10A**). The ionic signal intensity of ABA in the persistent calyx fruit was more uniformly distributed before, during, and after the formation of the abscission zone, and a stronger ABA ionic signal intensity and brighter color were observed during the formation of the abscission zone (**Figure 10B**). In addition, the results of relative quantification of the MALDI-MSI (**Figure 10C**) in the abscission zone of the decalyx fruit were significantly greater than those of the persistent calyx fruit (1.83, 2.84, and 1.67) before (2.11), during (3.169), and after the formation of the abscission zone (1.80). Additionally, the ABA in both the decalyx and persistent calyx fruits was significantly higher during the formation of the abscission zone than before the formation of the abscission zone, with increases of 33.4% and 35.5% respectively, and this was also significantly higher than in the later stages of the formation, with increases of 43.2% and 41.6%, respectively. In addition, the HPLC quantification results were essentially consistent with the MALDI-MSI relative quantitative analysis, indicating that the ABA content in the abscission zone of the decalyx fruits was significantly greater than that of the persistent calyx fruits before (1.67 mg/g·FW), during (2.57 mg/g·FW), and after (3.16 mg/g·FW) the formation of the abscission zone. In contrast, the ABA content after abscission zone formation (3.16 mg/g·FW) was significantly higher than before (1.67 mg/g·FW) and during (2.57 mg/g·FW) abscission zone formation, and, especially, significantly higher than that in the persistent calyx fruit after abscission zone formation (0.85 mg/g·FW). ABA content in the formation of the abscission zone of decalyx fruit was significantly higher than that of the persistent calyx fruit by 21.9%, 15.2%, and 271.8%, before, during, and after the formation of the abscission zone, respectively (**Figure 10D**).

#### 3.3.6 Visualization analysis of ACC

**Figure 11:**
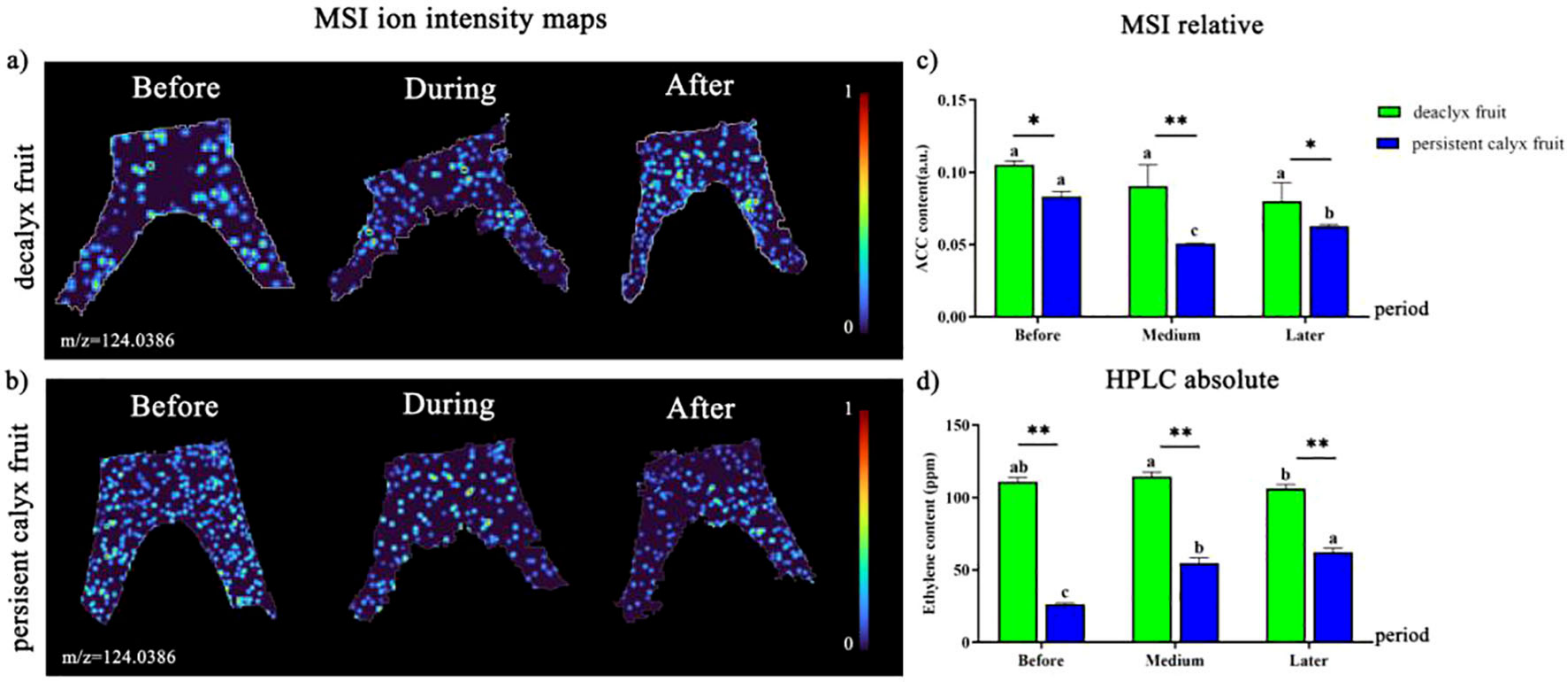
Visualized analysis of ACC during the calyx abscission process of Korla fragrant pear by MALDI-MSI and HPLC. **(A)** Mass spectrometry imaging of ACC ([M+Na]^+^, m/z 124.0386 m/z) in the abscission zone of the decalyx fruit calyx, and **(B)** mass spectrometry imaging of ACC ([M+Na]^+^, m/z 124.0386 m/z) in the corresponding abscission zone area of the persistent calyx fruit. **(C)** HPLC determination of the ETH content. **(D)** MALDI-MSI determination of ACC. A *t-test* was used to analyze the difference in phytohormone content in the abscission zone of the decalyx fruits and the corresponding part of the abscission zone of the persistent calyx fruits. *: Significant; **: Highly significant.

The distribution of ACC in the calyx tube site and related regions of the decalyx fruit and persistent calyx fruit significantly differed during the calyx abscission process (**Figure 11**). A brighter color indicated a stronger ionic signal strength of ACC. During abscission zone formation, the distribution of ACC ([M+Na]^+^, m/z 124.0386) in the abscission zone of the decalyx fruit and the corresponding part of the persistent calyx fruit was relatively limited before, during, and after the formation of the abscission zone. The distribution of ACC did not exhibit distinct regional patterns (**Figures 11A, B**). In addition, from the results of the relative MALDI-MSI quantification (**Figure 11C**), the ACC content of the abscission zone in the decalyx fruit calyx gradually decreased. However, the ACC content of the corresponding part of the abscission zone in the persistent calyx fruit tended to decrease and then increase. In addition, the results from the HPLC quantification were basically consistent with the results from the relative quantification by MALDI-MSI. The content of ETH was significantly greater at the stage of abscission zone formation (114.36 ppm) than after the formation of the abscission zone (106.24 ppm), and the corresponding part of the abscission zone of the persistent calyx fruits showed a tendency towards a gradual increase in the content of ETH, which reached a maximum after abscission zone formation. ETH content in the formation of the abscission zone of the decalyx fruit was significantly higher than that of the persistent calyx fruit by 25.0%, 80.0%, and 26.9% before, during, and after the formation of the abscission zone, respectively (**Figure 11D**).

## 4 Discussion

### 4.1 Characteristics of calyx abscission and the development process of the abscission zone in Korla fragrant pear

Plant organ abscission is a common phenomenon, referring to the physiological process by which plant tissues or organs separate from the parent body, that is, the formation of the abscission zone and the separation of the abscission layer (Patharkar and Walker, 2018; Yu et al., 2020). In this study, it was found that during the formation process of the abscission zone in the decalyx fruit calyx, a distinct yellow ring-abscission zone can be observed at the junction of the calyx and the young fruit, which was an important basis for determining whether the calyx of Korla fragrant pear had abscinded (Hong et al., 2011). The ultrastructure of the cells in the abscission zone during plant organ abscission had unique structural characteristics (Patharkar and Walker, 2018). Observation of the ultrastructural features of the calyx abscission zone in Korla fragrant pear revealed that during the formation of the abscission zone, the persistent calyx fruit had more chloroplasts than the abscission zone in the decalyx fruit calyx, accompanied by the production of starch granules. After the formation of the abscission zone, a distinct membrane layer outside the walls of the cells in the abscission zone in the decalyx fruit was observed, whereas in the persistent calyx fruit abscission zone cells, the organelles remained intact, and endoplasmic reticulum was produced within the cells. Based on the results from this study, the causes of calyx abscission were hypothesized to be the following: first of all, chloroplasts, which are the site of photosynthesis, could provide energy for calyx growth and development. When photosynthesis was vigorous, the photosynthesis products that could not transported out in time were converted into starch granules. At the same time, the cells in the corresponding parts of the abscission zone of the persistent calyx fruit contained a large number of chloroplasts, which is caused by vigorous photosynthesis. As a result, the accumulation of starch granules increased, providing an excess of energy required for calyx development, which promoted the retention of the calyx in the persistent calyx fruits (Gallei et al., 2020). Second, the endoplasmic reticulum is closely related to the synthesis of lipids, sugars, and phytohormones, and the appearance of the endoplasmic reticulum after abscission zone formation may be accompanied by a release of energy from sugars or the synthesis of phytohormones, which indirectly or directly contribute to the calyx development (Wang, 2008). On this basis, we hypothesized that the reason for calyx abscission in Korla fragrant pear was most likely due to physiological abscission caused by its own physiological activities, rather than normal abscission caused by senescence or maturity, or stress abscission caused by external stimuli. It can be seen that during the process of abscission zone formation in the calyx of Korla fragrant pear, energy metabolism played an important role. However, there is currently little research on the regulatory mechanism of calyx abscission in terms of nutritional substances, mineral elements, and other factors. Therefore, future research can further improve the study of the regulatory mechanism of calyx abscission in Korla fragrant pear from the perspective of materials and energy metabolism.

### 4.2 Changes in plant hormone content and spatial distribution characteristics during calyx abscission in Korla fragrant pear

High concentrations of IAA have been reported to inhibit plant organ abscission (Raven, 1975). In the persistent calyx fruit of Dangshan Su pear 10 days after full bloom, the genes related to calyx abscission were significantly upregulated compared to the decalyx fruit. Moreover, most IAA transcription factors were highly expressed in the persistent calyx fruit, leading to a significant increase in indole-3-acetaldehyde oxidase activity (IAAldO), thereby accelerating the synthesis of IAA and inhibiting the occurrence of calyx abscission (Guo et al., 2024), which was consistent with the results of this study. Mass spectrometry imaging showed that the signal intensity of IAA ions in the corresponding part of the abscission zone of the persistent calyx fruit was greater than that of the abscission zone of the decalyx fruit, and its color was brighter. In addition, the IAA content within the persistent calyx fruit was significantly greater than that of the decalyx fruit, and it reached a maximum during the formation of the abscission zone. Moreover, HPLC quantitative analysis also confirmed these results. This likely occurred because the auxin stimulated the development and formation of the vascular bundle tissues by promoting the elongation and division of the cells in the persistent calyx fruit, and accelerated the transport of IAA to the distal axial end of the calyx abscission zone, which reduced the sensitivity of the cells to ETH, thus inhibiting calyx abscission (Raven, 1975; Li et al., 2022). Therefore, IAA plays an important role in inhibiting calyx abscission in Korla fragrant pear.

It is currently widely believed that GA_3_ affects organ abscission directly or indirectly through interactions with other phytohormones. High levels of GA_3_ and IAA in the calyx of Korla fragrant pear have been reported to be important reasons for calyx persistence (Ma et al., 2019). In addition, exogenous application of GA to navel oranges significantly promoted the accumulation of endogenous GA, IAA, and ZR, slowed down the decline rate of endogenous GA, IAA, and ZR, and thereby inhibited the abscission of fruits (Ren et al., 2023; Xiong et al., 2012). Visualization of the calyx abscission zone revealed that GA_3_ was enriched in the corresponding part of the persistent calyx fruits, and the GA_3_ content in the corresponding part of the persistent calyx fruit was significantly higher than that in the abscission zone of the decalyx fruit, which was consistent with the relative quantitative results of HPLC. These results indicated that GA_3_ inhibited calyx abscission, and it is speculated that the reason may be that high concentrations of GA_3_ can promote the biosynthesis of auxins and inhibit the accumulation of ACC, thereby reducing the sensitivity of the abscission zone to ETH and delaying abscission (Marciniak et al., 2018).

The impact of ZT on plant organ abscission is relatively minor, but since ZT is a type of CTK that delays ACC biosynthesis and thus inhibits ETH synthesis, ZT causes a reduced sensitivity of the abscission zone to ETH, indirectly affecting organ abscission (Tay et al., 1986). Our results showed that ZT was concentrated in the corresponding parts of the abscission zone of the persistent calyx fruits before and during the formation of the abscission zone, whereas the abscission zone of ZT in the decalyx fruits was enriched after the formation of the abscission zone. Moreover, the relative quantitative results from MALDI-MSI and HPLC are consistent with those from visualization analysis. Notably, the ZT content in the persistent calyx fruit was significantly greater than that in the decalyx fruit before and during abscission zone formation, which indicated that ZT mainly plays a role before and during the formation of the abscission zone in the process of calyx abscission.

ETH is the main phytohormone that controls the senescence and abscission of plant organs. On the one hand, high concentrations of ETH could promote tomato flower abscission (Sundaresan et al., 2018). On the other hand, exogenous spraying of 1-methylcyclopropene (1-MCP, an ethylene perception inhibitor) inhibited the polygalacturonase activity, thereby delaying tomato fruit abscission (Chersicola et al., 2017). This study found that during the calyx abscission process, visual analysis showed that the distribution of ACC in the abscission zone of the decalyx fruit and the corresponding part of the abscission zone of the persistent calyx fruit did not exhibit obvious regionality. The ACC content in the abscission zone of the decalyx fruit was significantly higher than that in the persistent calyx fruit, which was basically consistent with the results of the relative quantitative determination of ETH content by HPLC. This may be due to the fact that ETH itself acted as a promoter of organ abscission, promoted the synthesis of hydrolytic enzymes such as cellulase, pectinase and other enzymes in the induced abscission zone, and controlled the release of cellulase from the protoplast into the cell wall, thus promoting cell senescence and cell wall breakdown, which ultimately leads to the abscission of the plant organs (Wang et al., 2002; Yasong et al., 2018; Deng et al., 2023). It is worth mentioning that ACC, as an intermediate in the conversion of S-adenosylmethionine (SAM) to ETH, was converted into ethylene through an enzyme-catalyzed reaction. Generally, ETH acted directly at the site of its synthesis (Yu et al., 1979; Wang et al., 2002).

ABA is an important phytohormone for inducing organ abscission and can directly or indirectly stimulate ethylene biosynthesis (Zhao et al., 2020). The transcriptional activities of ACC synthase (LlACS) and ACC oxidase (LlACO) were significantly increased in yellow lupine flowers treated with ABA, which increased the level of the 1-aminocyclopropane-1carboxylic acid-ethylene precursor, leading to the onset of abscission (Wilmowicz et al., 2016). Our results showed that ABA accumulated extensively and was widely distributed in the abscission zone of the decalyx fruit calyx, and the ABA content in the decalyx fruit calyx was significantly higher than that in the persistent calyx fruit. Moreover, the relative quantitative results from MALDI-MSI and HPLC were relatively consistent with the results of the visual analysis. Thus, it can be seen that high ABA levels in the calyx abscission zone could promote calyx abscission, which is potentially because ABA inhibited the polar transport of IAA or enhanced the enzyme activity of ACO and ACS to promote ETH synthesis. At the same time, a high concentration of ABA promoted the synthesis of cell wall-degrading enzymes, thereby stimulating the abscission zone cells to receive the abscission signal and inducing the abscission of plant organs (Anfang and Shani, 2021; Qureshi et al., 2021).

This study conducted a relative quantitative analysis of plant hormones in the calyx of the abscission zone of the Korla fragrant pear using MALDI-MSI technology. The higher the quantitative value, the higher the content of the target substance in that area, which was consistent with Wang’s application of MALDI-MSI technology to determine the distribution of plant metabolites in strawberries (Wang et al., 2021). Furthermore, to further verify the rationality and validity of the data, we used HPLC and GC to perform absolute quantification of plant hormones in the calyx of the abscission zone, confirming that the distribution of plant hormones in the calyx of the abscission zone is not uniform but concentrated in the abscission zone area. This finding revealed the spatial distribution of plant hormones in the abscission zone during the flower calyx abscission process in Korla fragrant pear. However, the regulatory network for calyx abscission is complex and diverse. Therefore, future research integrating energy metabolism, internal hormones, nutrients, mineral elements, and the abscission-related enzymes of the calyx could contribute to a comprehensive understanding of the mechanism of calyx abscission in Korla fragrant pear.

## 5 Conclusion

The external morphological features and cellular ultrastructures of the decalyx and persistent calyx fruit during calyx abscission in Korla fragrant pear were clearly different. First, a distinct yellow ring-abscission zone can be clearly observed at the junction of the young fruit in the decalyx fruit. Second, regarding the cellular ultrastructure, the mitochondria in the abscission zone of the decalyx fruits were regularly distributed around the cell walls with a moderate amount of chloroplasts, whereas in the corresponding part of the abscission zone in the persistent calyx fruits, the mitochondria were scattered throughout the cells with a higher number of chloroplasts, which contain starch granules within them.

ABA and ACC were enriched in the abscission zone of the decalyx fruits, and the results from MALDI-MSI and HPLC also confirmed that the content of ABA and ETH in the decalyx fruit abscission zone was significantly higher than that in the corresponding part of the abscission zone of the persistent calyx fruit, indicating that ABA and ETH promote calyx abscission. In contrast, IAA and GA_3_ were concentrated in the corresponding parts of the abscission zone of the persistent calyx fruit, and their contents were significantly higher than those in the abscission zone of the decalyx fruit, indicating that IAA and GA_3_ inhibited calyx abscission. A regulatory effect of ZT on calyx abscission was not obvious.

## Data Availability

The raw data supporting the conclusions of this article will be made available by the authors, without undue reservation.
