# Supplementary material for: Phytohormonal dynamics in the abscission zone of Korla fragrant pear during calyx abscission: a visual study
Source: Front Plant Sci. 2024 Oct 8;15:1452072. doi: 10.3389/fpls.2024.1452072 (PMC11493647; doi:10.3389/fpls.2024.1452072)
Supplement: Supplementary file 1 [file DataSheet1.doc]

Supplemental files

Table S1: Standard Substance Information

| Compound | Firm | Product number | CAS |
| --- | --- | --- | --- |
| Abscisic acid | Katchem，Czech Republic | OIC-0132721 | 21293-29-8 |
| Ethylene | Sigma，Germany | A3903-100MG | 22059-21-8 |
| trans-Zeatin | Leyan，China | / | 6025-53-2 |
| Gibberellin A3 | Katchem，Czech Republic | 0122501 | 77-06-5 |
| Indole-3-acetic acid | Katchem，Czech Republic | OIC-0031531 | 87-51-4 |

Table S2: Reagents and instrument information

| Name | Purity | Firm |
| --- | --- | --- |
| Acetonitrile | Suitable for HPLC | Merck, Germany |
| 2,5-dihydroxybenzoic acid (DHB) | ≥99.0% | Sigma, Germany |
| Tims TOF Flex MS System | - | Bruker Daltonics, Germany |
| TM-Sprayer (Matrix Sprayer) | - | Bruker Daltonics, Germany |
| CM1950-Slicing Machine | - | Leica, Germany |
| Pipette | - | Eppendorf, Germany |
| ITO Conductive Slide | - | Gulo Glass, China |
| MIX-200 Multitube Vortex Oscillator | - | ShangHai JinXin, China |
| KQ5200E Ultrasonic Cleaner | - | KunShan ShuMei, China |


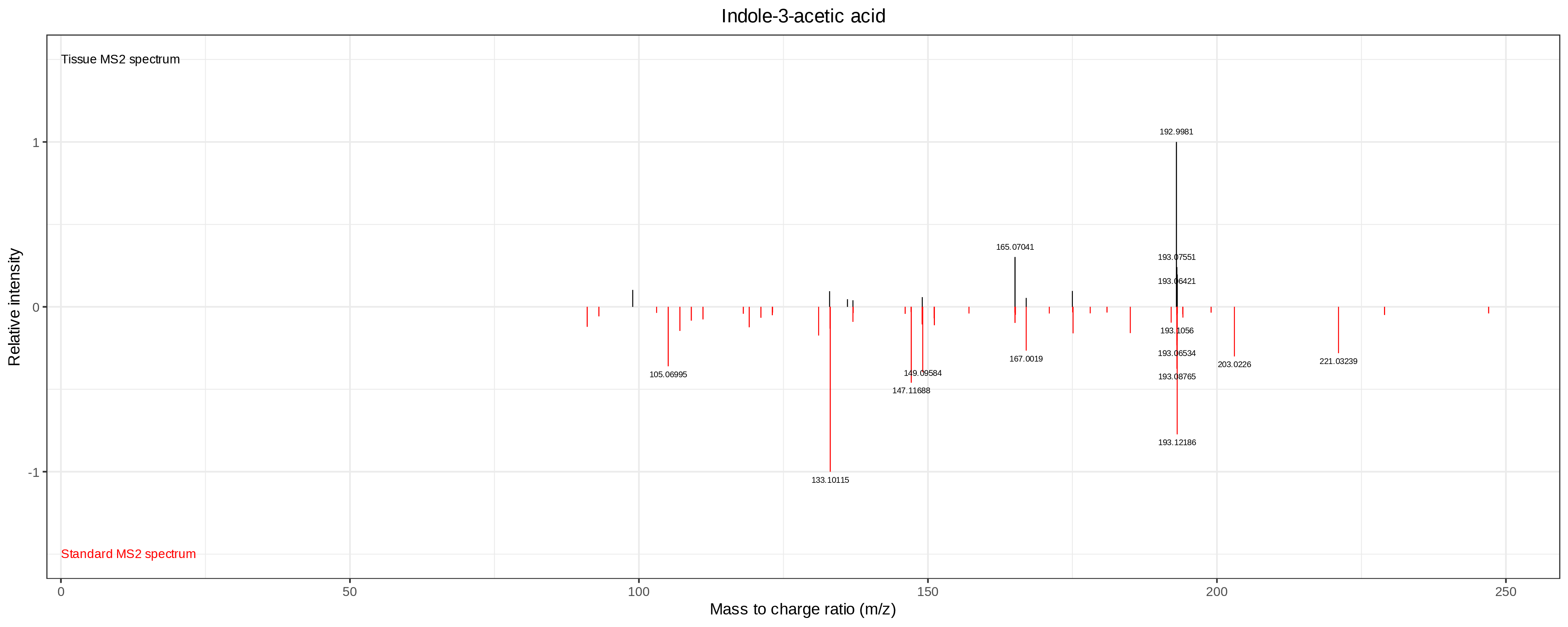


Figure S1: The secondary mass spectrum of IAA


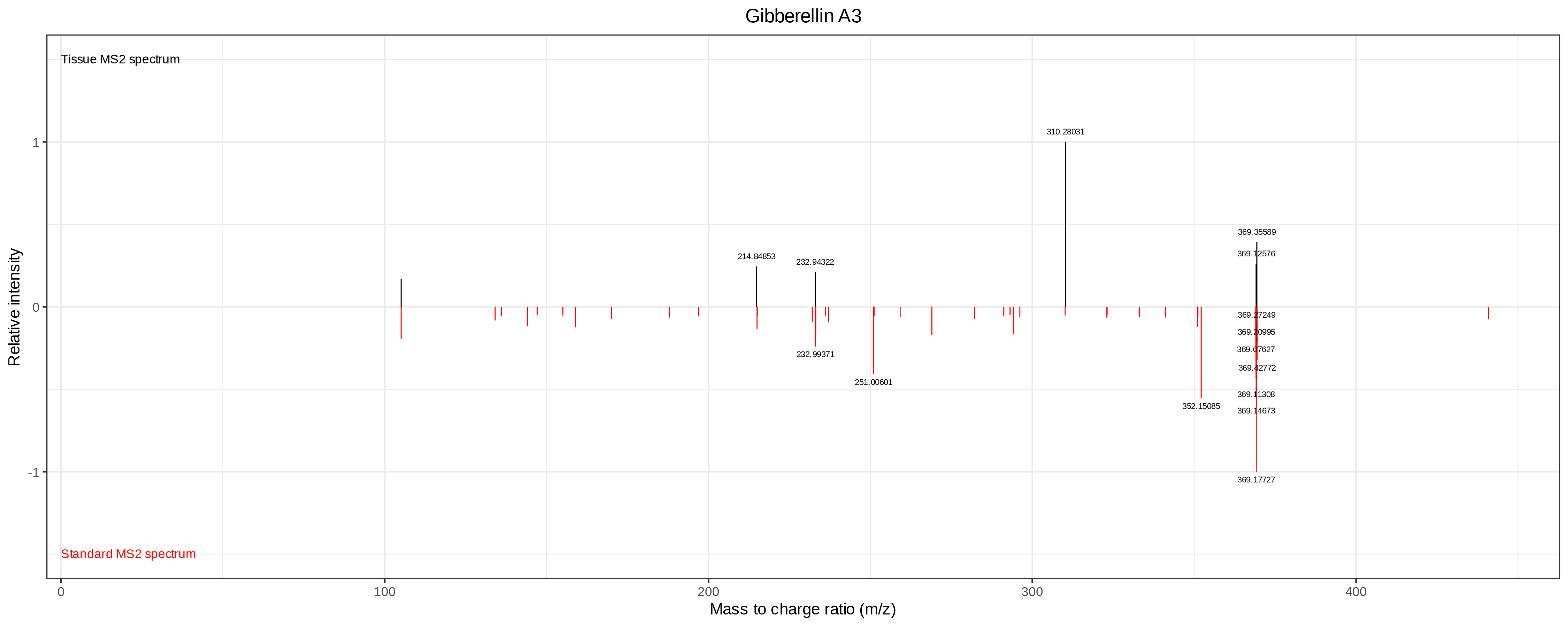


Figure S2: The secondary mass spectrum of GA3


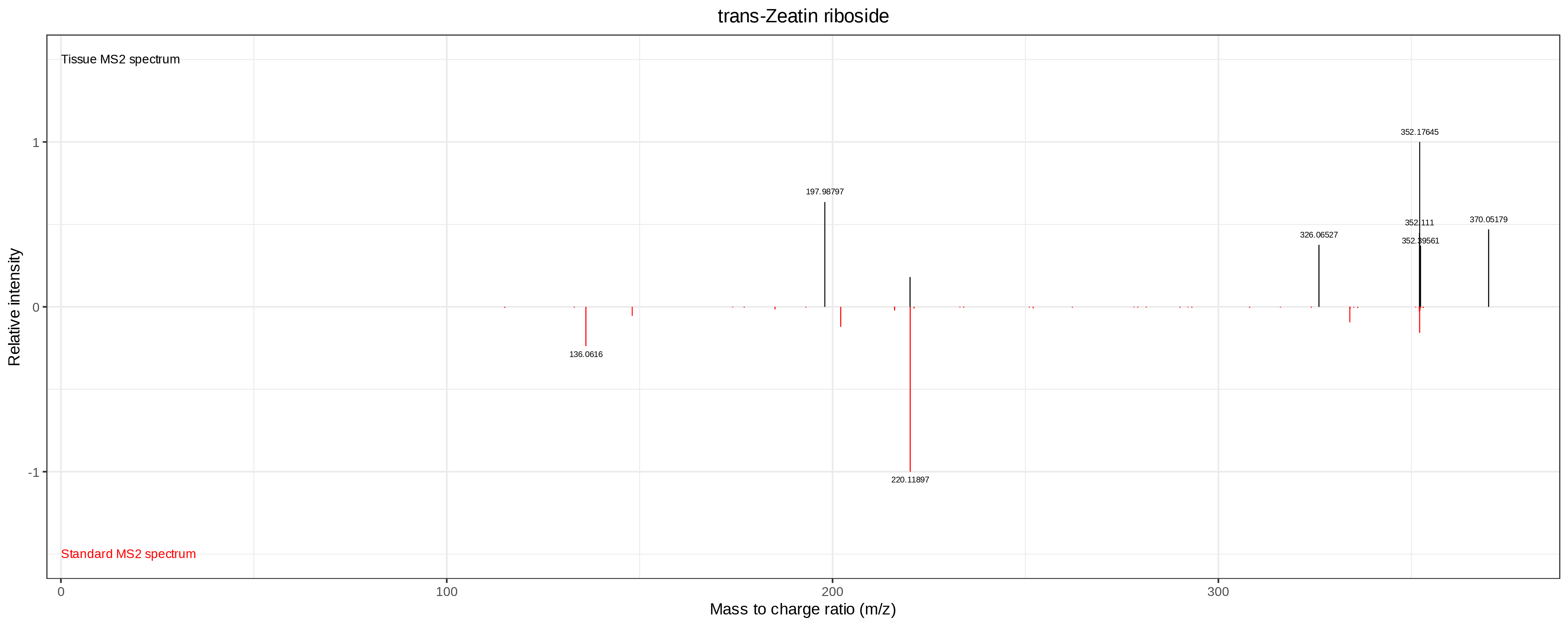


Figure S3: The secondary mass spectrum of ZT


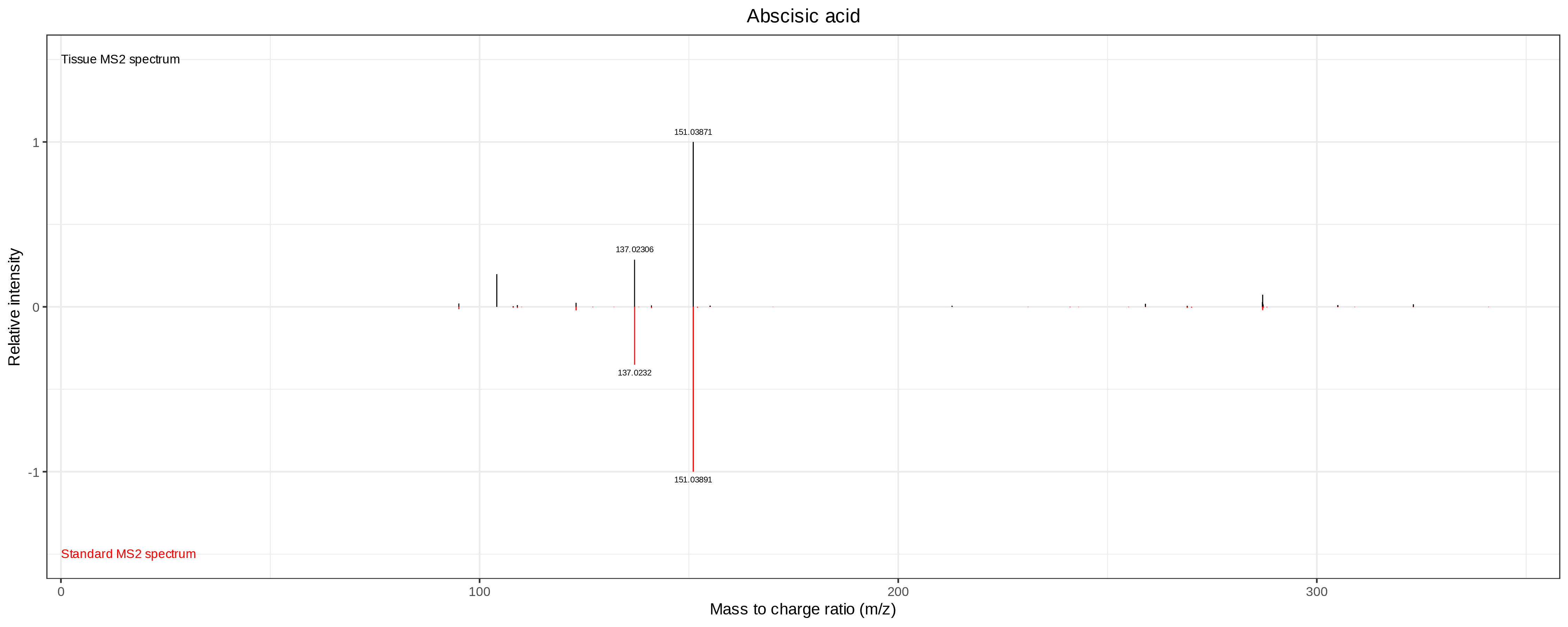


Figure S4: The secondary mass spectrum of ABA


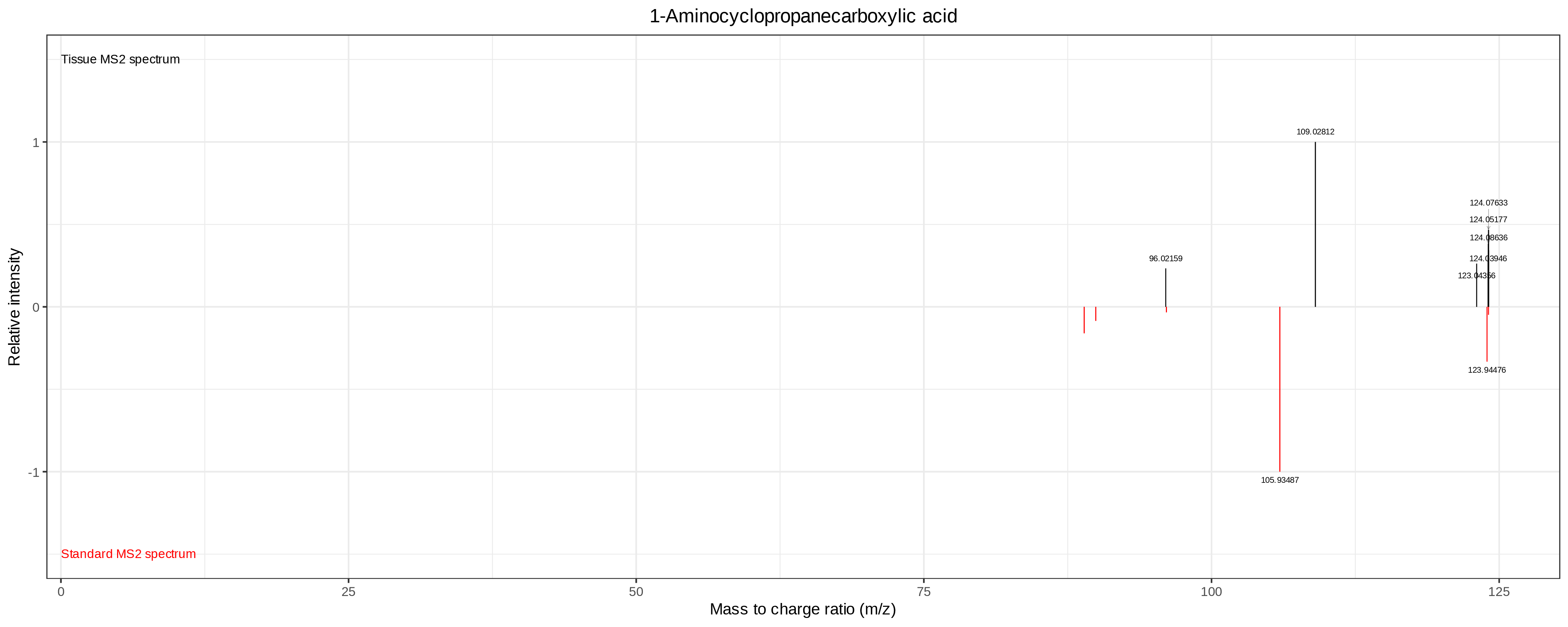


Figure S5: The secondary mass spectrum of ACC


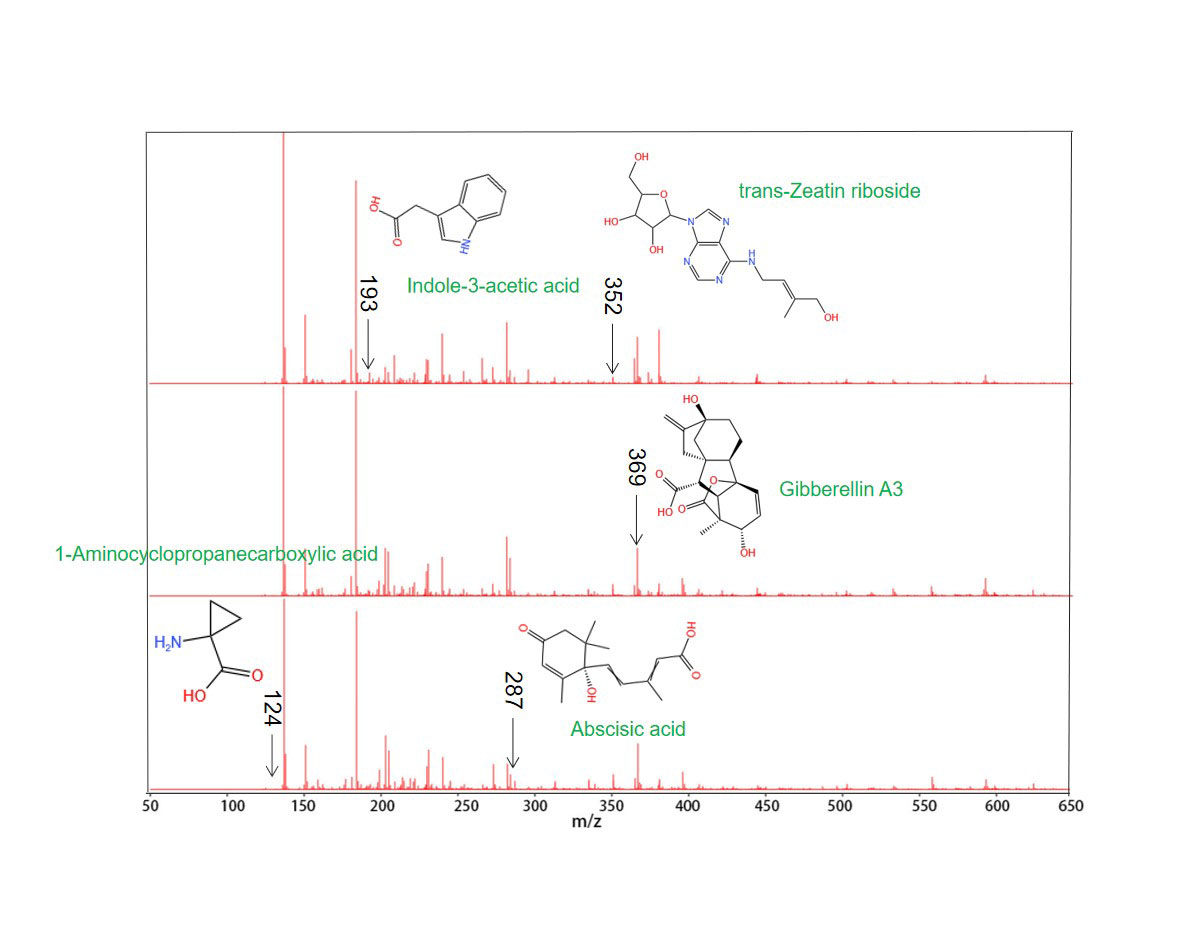


Figure S6: MALDI-TOF average mass spectrum of tissue sections in the calyx abscission zone during the calyx abscission process of Korla fragrant pear


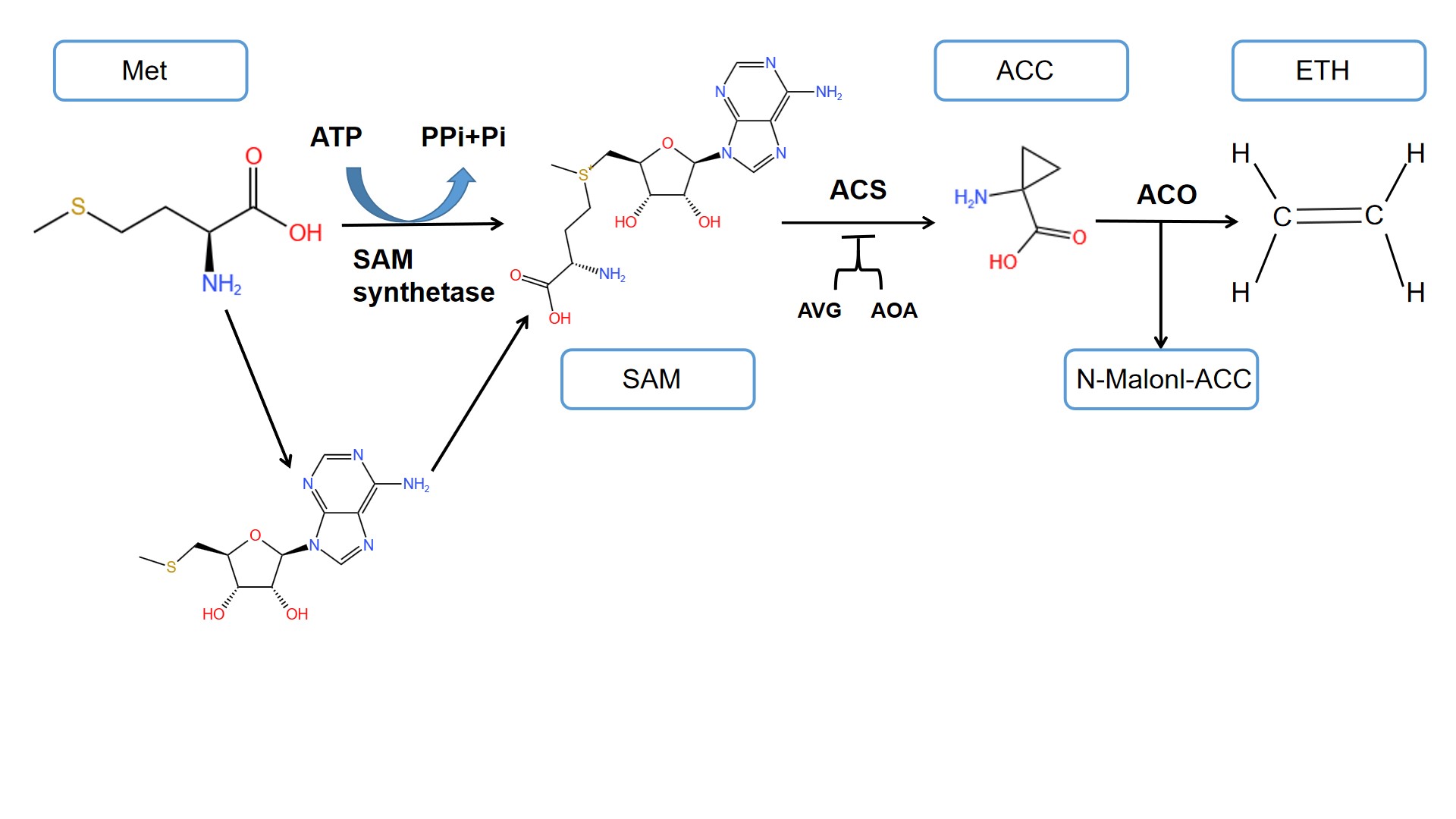


Figure S7: Biosynthetic pathway of ETH
